# Diesel exposure increases susceptibility of primary human nasal epithelial cells to rhinovirus infection

**DOI:** 10.14814/phy2.14994

**Published:** 2021-09-20

**Authors:** Loretta Müller, Jakob Usemann, Marco P. Alves, Philipp Latzin

**Affiliations:** ^1^ Division of Paediatric Respiratory Medicine and Allergology Department of Paediatrics, Inselspital Bern University Hospital University of Bern Bern Switzerland; ^2^ Department for BioMedical Research (DBMR) University of Bern Bern Switzerland; ^3^ University Children's Hospital Basel (UKBB) Basel Switzerland; ^4^ Division of Respiratory Medicine University Children's Hospital Zurich Zurich Switzerland; ^5^ Institute of Virology and Immunology Bern Switzerland; ^6^ Department of Infectious Diseases and Pathobiology Vetsuisse Faculty University of Bern Bern Switzerland

**Keywords:** adults, air pollution, air–liquid‐interface cell culture, children, respiratory infection

## Abstract

Nasal epithelial cells (NECs) are among the first cells to be exposed to air pollutants and respiratory viruses. Although it is known that air pollution exposure and rhinovirus infections increase the risk for asthma development independently, it is unclear how these risk factors interact on a cellular level. Therefore, we aimed to investigate how exposure to diesel particulate matter (DPM) modifies the response of primary NECs to rhinovirus (RV) infection in vitro. Exposure of re‐differentiated, primary NECs (49 healthy children [0–7 years], 12 adults) to DPM modified the mRNA expression of viral cell‐surface receptors, pattern recognition receptors, and pro‐inflammatory response (also protein levels). After exposure to DPM, we additionally infected the NECs with RV‐1b and RV‐16. Viral loads (assessed by titration assays) were significantly higher in DPM‐exposed compared with non‐exposed NECs. Exposure to DPM prior to RV infection resulted in a significant upregulation of pro‐inflammatory cytokines (mRNA and protein level) and β‐defensins mRNA, and significant downregulation of pattern recognition receptors mRNA and CXCL10 (mRNA and protein levels). There was no difference between all outcomes of NECs from children and adults. We can conclude that exposure to DPM prior to RV infection increases viral loads by downregulation of viral defense receptors and upregulation of pro‐inflammatory cytokines. Our findings indicate a strong interaction between air pollution and the antiviral response to RV infection in NECs. We provide mechanistic evidence that exposure to air pollution increases susceptibility to RV infection.

## Graphical abstract

Exposure to diesel exhaust particles change the levels of immune factors and increase the susceptibility of respiratory epithelial cells to rhinovirus infections.

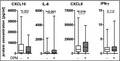

## 1 INTRODUCTION

Air pollution is associated with severe adverse health effects including oncological, cardiovascular, and respiratory diseases (Anderson et al., 2012; Liu & Grigg, 2018). Although air pollution‐related deaths are reported mainly in older adults, there is growing concern that air pollution has major adverse effects in children, including long‐term consequences on respiratory health (Chen et al., 2015; Usemann et al., 2018). In urban areas, diesel engines—and traffic in general—contribute substantially to air pollution (Karaguliana et al., 2015). One of the first cell types exposed to air pollutants are respiratory epithelial cells (Muller et al., 2010; Mullins et al., 2016). This has led to studies showing an increase in inflammatory response, higher oxidative stress (Kumar et al., 2015; Muller et al., 2010; Mullins et al., 2016; Zarcone et al., 2016, 2018) and reduced epithelial barrier function (Zarcone et al., 2016) after respiratory epithelial cells are exposed to diesel exhaust (DE).

Besides responding to environmental pollutants, another important role of respiratory epithelial cells is protection against viral infection and the regulation of immune responses and inflammation (Muller & Jaspers, 2012; Whitsett & Alenghat, 2015). A relevant virus causing respiratory infection in children and adults is the human rhinovirus (RV). RV infections are responsible for more than half of cold‐like illnesses and are the most common causes of upper respiratory tract infections (Jacobs et al., 2013). They play a major role in respiratory morbidity, wheezing illnesses of children (Heymann et al., 2004; Kieninger et al., 2013; Korppi et al., 2004; Piotrowska et al., 2009), and asthma development and exacerbations (Jackson et al., 2008; Williams et al., 2021). Infection of the respiratory mucosa with RV occurs via cell‐surface receptors, including the intracellular adhesion molecule 1 (ICAM‐1, for RVs of the major group, such as RV‐16) and the low density lipoprotein receptor (LDLR, for RVs of the minor group, such as RV‐1b; Palmenberg et al., 2009). Infected nasal epithelial cells (NECs) sense RV via pattern recognition receptors (PRR; e.g., toll‐like receptors [TLRs], retinoic acid‐inducible gene I [RIG‐I] or melanoma differentiation‐associated protein 5 [MDA5]; Stokes et al., 2016). Subsequently, defensins (Proud et al., 2004), interferons (IFNs), and immune regulatory chemokines, and cytokines (e.g., C‐X‐C motif chemokine 10 [CXCL10], interleukin [IL] 1β, IL6 and CXCL8) are released and initiate the immune response (Yeo et al., 2010).

Only a few studies have investigated how air pollution affects the antiviral responses of airway cells (Muller & Jaspers, 2012; Takizawa, 2004). It has been shown that DE emissions enhanced viral replication and the release of IFN‐γ in airway epithelial cells in vitro and in vivo after influenza infection (Jaspers et al., 2005; Noah et al., 2012). Previous studies were limited by the low number of subjects investigated and were conducted in adults only. However, investigating the antiviral response in children is crucial due to their differing antiviral response compared with adults (Usemann et al., 2020). Further, most previous studies have investigated the effects in cell lines, which are poor surrogates of the native respiratory epithelium (Zarcone et al., 2016).

Our aim was to study the modifying effect of diesel exposure on RV infection in primary NECs of children and adults. We hypothesize that similar to the previously described pro‐inflammatory effect of diesel exposure on influenza infection (Jaspers et al., 2005; Noah et al., 2012), diesel exposure prior to RV infection results in higher levels of viral load and pro‐inflammatory immune response, and modifies immune factor expression.

## 2 MATERIAL AND METHODS

### 2.1 Study design

We cultured NECs from children and adults to compare the response to RV‐16 and RV‐1b infection between diesel particulate matter (DPM)‐exposed cells and non‐DPM‐exposed, but also infected controls. Outcome parameters were viral load and levels of various immune factors of the early epithelial immune response.

**FIGURE 1:**
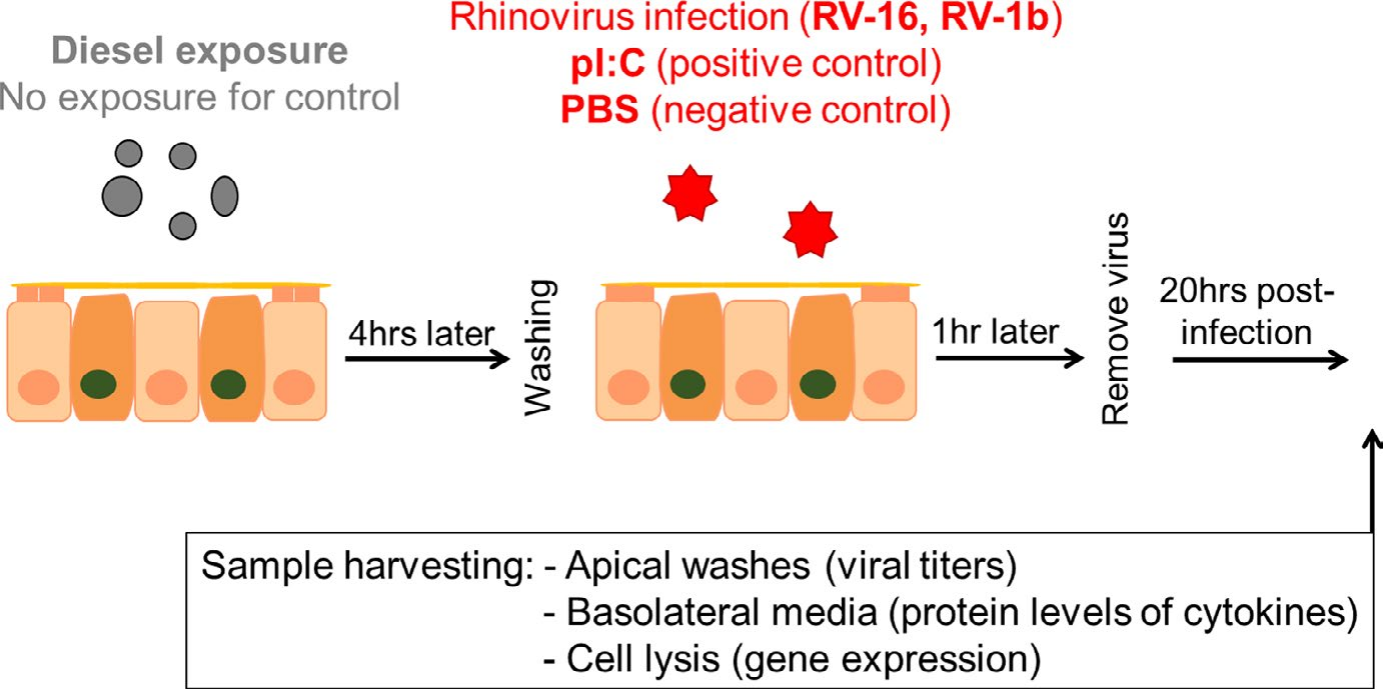
Experimental setup. Re‐differentiated nasal epithelial cell cultures were exposed to diesel particulate matter (no exposure for controls) and 4 h later infected with two types of rhinovirus (pI:C infection as a positive control, phosphate‐buffered saline [PBS] only for negative control). One hour later the virus was removed and 20 h post‐infection the apical washes, basolateral media and the cells were harvested. For an overview about the experimental matrix, see Figure S1 in the online supplement

NECs were obtained via nasal brushings from healthy, non‐asthmatic, and non‐smoking adult volunteers and from children without any respiratory problems undergoing elective surgery (Stokes et al., 2014; Usemann et al., 2020). Children were all non‐asthmatic and not exposed to considerable levels of passive smoke. After cultivation and re‐differentiation, NECs were exposed to DPM (no exposure for non‐DPM controls). Four hours later, NECs were infected with RV‐16, RV‐1b, or polyinosinic:polycytidylic acid (pI:C; positive control; no infection for non‐infected controls). At 20‐h post‐infection, at the peak of RV replication (Nakagome et al., 2014; Sykes et al., 2014), we harvested apical washes, basolateral supernatants and cell lysates and stored them at −80℃ for later analysis. Figures 1 and S2 show the experimental setup and matrix. We used one transwell per participant for every condition, thus cells from the same donors were used for all experimental groups.

The study was approved by the Ethics Committee Nordwest‐ und Zentralschweiz, Switzerland (reference number 250/13). We obtained written informed consents from all participants.

The study was originally designed to study two aims: (1) age‐dependency of RV infections and (2) impact of DPM exposure on RV infection. Since access to primary NECs from children is very limited, we addressed both aims with one experimental setup. However, to avoid overloading one publication, we decided to split the data for two separate articles. Thus, we have had to use parts of the data for both publications. Concretely, this means that the data without DPM exposure have already been published to study the age‐dependency of RV infections (Usemann et al., 2020), but are used in this publication as non‐DPM controls.

### 2.2 Cell cultures

Primary NECs were cultured using the PneumaCult Media (Stemcell Technologies) according to the manufacturer's protocol with minor changes indicated in the Supporting Information and as previously published (Usemann et al., 2020).

### 2.3 DPM exposure and virus infection

We sprayed standard diesel particulate matter (DPM; National Institute of Standards and Technology (NIST), cat# 1650b) as a model air pollutant on the nasal epithelial cells (NECs) using a Dry Powder Insufflator™ (PennCentury; Figure S2). Briefly, approximately 300 µg DPM was weighed in a 0.5 ml low retention Eppendorf tube and transferred into the loading chamber. One puff (volume of 200 µl) of the insufflator was sprayed on the NECs what deposited approximately half of the amount loaded, thus approximately 150 µg DPM on the cell cultures (see Figure S3). We found no cytotoxicity for this dose (Figure S4).

Four hours after spraying DPM on the NECs, we rinsed them once with phosphate‐buffered saline with magnesium and calcium (PBS; Sigma–Aldrich) and subsequently infected them. NECs were infected using RV‐16 (major group) and RV‐1b (minor group) both at a multiplicity of infection (MOI) 1 and 4. The virus was added in a final volume of 100 μl in PBS to the apical side of the cell culture for 1 h at 37℃. The viral mimetic pI:C (10 μg/ml) and PBS alone were used as positive and negative controls, respectively.

### 2.4 Analysis of virus titration, quantitative real‐time RT‐PCR, protein measurement

Viral load was determined using the TCID50 method (according to Schogler et al., 2015; more details are given in the Supporting Information). RNA extraction (using RNA Clean & Concentrator‐5 w/Zymo‐Spin IC Columns; Zymo Research), cDNA synthesis (using 200 ng of total RNA and the GoScript Reverse Transcription System; Promega, cat.no. A5003), quantitative real‐time RT‐PCR (384‐well plates, duplicates for target genes, quadruplicates for the housekeeping gene; GoTaq qPCR Master Mix system, (Promega, cat.no. A6002) were performed according to manufacturer's instructions (details are given in the Supporting Information). Protein levels were measured using a human cytokine/chemokine magnetic bead panel (Milliplex MAP kit, Millipore/Merck), a Magpix Luminex instrument and the xPONENT software (version 4.2, Luminex Corp) according to manufacturer's instructions (single measurements and overnight incubation at 4℃; more details are given in the Supporting Information).

### 2.5 Statistics

Comparison of outcomes between DPM‐exposed and non‐exposed cell cultures was done using the paired Wilcoxon‐signed‐rank test, and comparison between outcomes of cell cultures from children and adults after DPM exposure (values normalized to non‐exposed controls) was done using the Mann–Whitney test (STATA [version 16], STATA Corp). A *p* < 0.05 defined statistical significance.

## 3 RESULTS

### 3.1 Study population

We included 49 children (mean age [range] = 3.4 [0.21–7.7] years) and 12 adults (35.1 [24.9–65.6] years).

### 3.2 DPM exposure modifies the expression of immune factors

**FIGURE 2:**
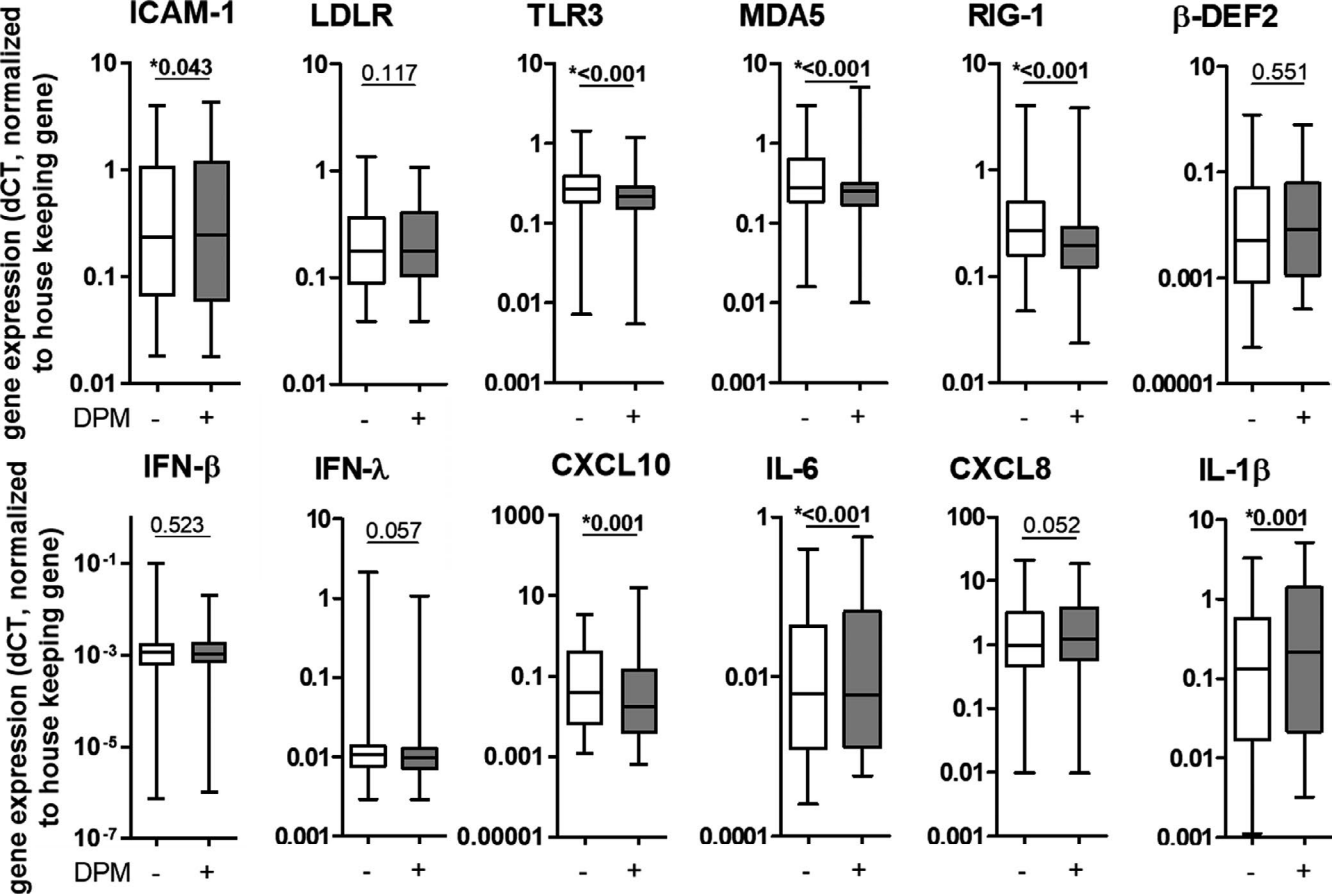
Diesel particulate matter exposure modifies immune factors in nasal epithelial cells on mRNA levels. Gene expression levels of different immune factors were analyzed 20 h post‐infection. Data are shown as a box and whisker plot with line at median, interquartile range (box) and range (line). *N* = 61, *p < 0.05, tested with Wilcoxon rank‐sum test

**FIGURE 3:**
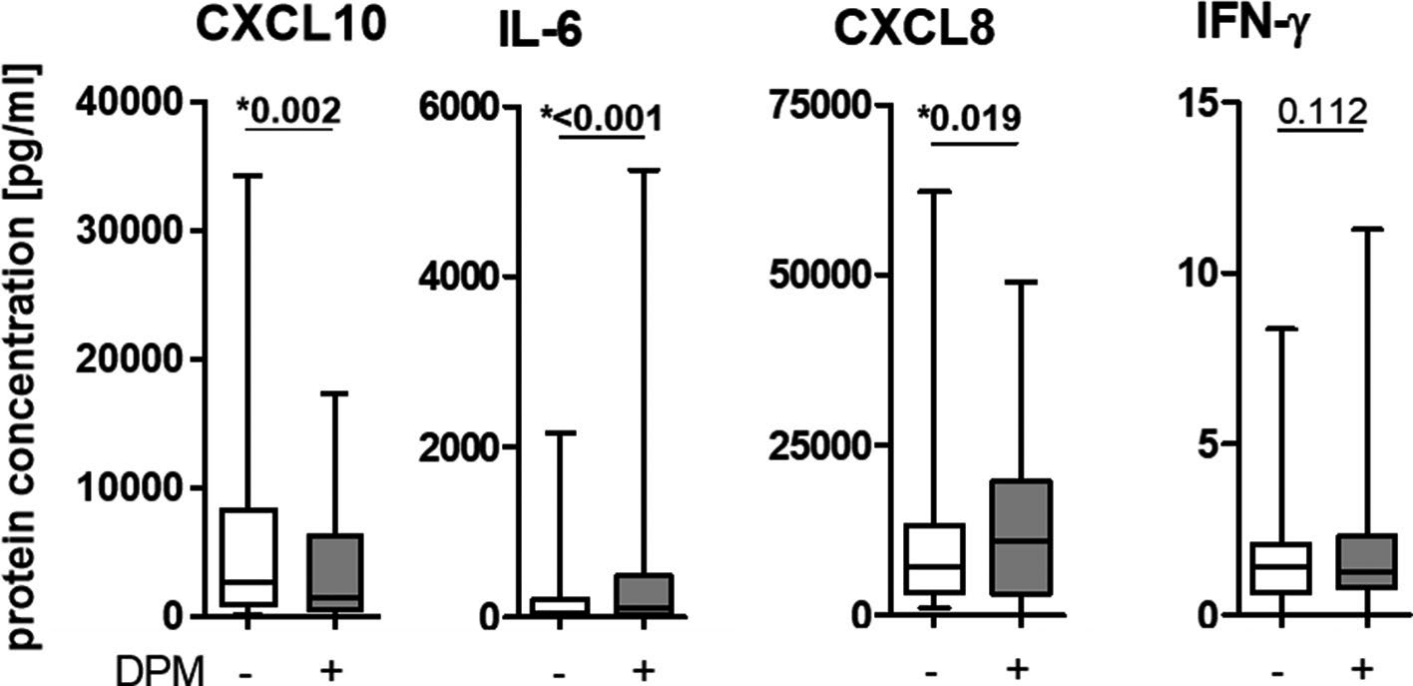
Diesel particulate matter exposure changes protein concentrations in the basolateral media of nasal epithelial cells. Protein levels of CXCL10, IL6, CXCL8, and IFN‐γ were assessed 20 h post‐infection using multiplex bead‐based immunoassays. Data are shown as a box and whiskers plot with line at median, interquartile range (box), and range (line). *N* = 60, *p < 0.05, tested with Wilcoxon rank‐sum test

DPM exposure of NECs significantly increased mRNA levels of the RV's cell‐surface receptor *ICAM1*, but not the *LDLR* receptor. DPM exposure significantly decreased expression of PRRs, namely *TLR3*, *MDA5*, and *RIG*‐*I*, and did not impact the mRNA levels of *β‐defensin 2* (*β*‐*def2)*, *IFN*‐*β*, and *IFN*‐*λ*. The immune response was modified by DPM exposure by significantly decreasing mRNA and protein levels of CXCL10 and increasing mRNA and protein levels of IL‐6 and CXCL8 (mRNA was only trendwise significant) and mRNA levels of *IL1β* (Figures 2 and 3, for details see Tables S4 and S5).

### 3.3 DPM exposure increases viral loads

**FIGURE 4:**
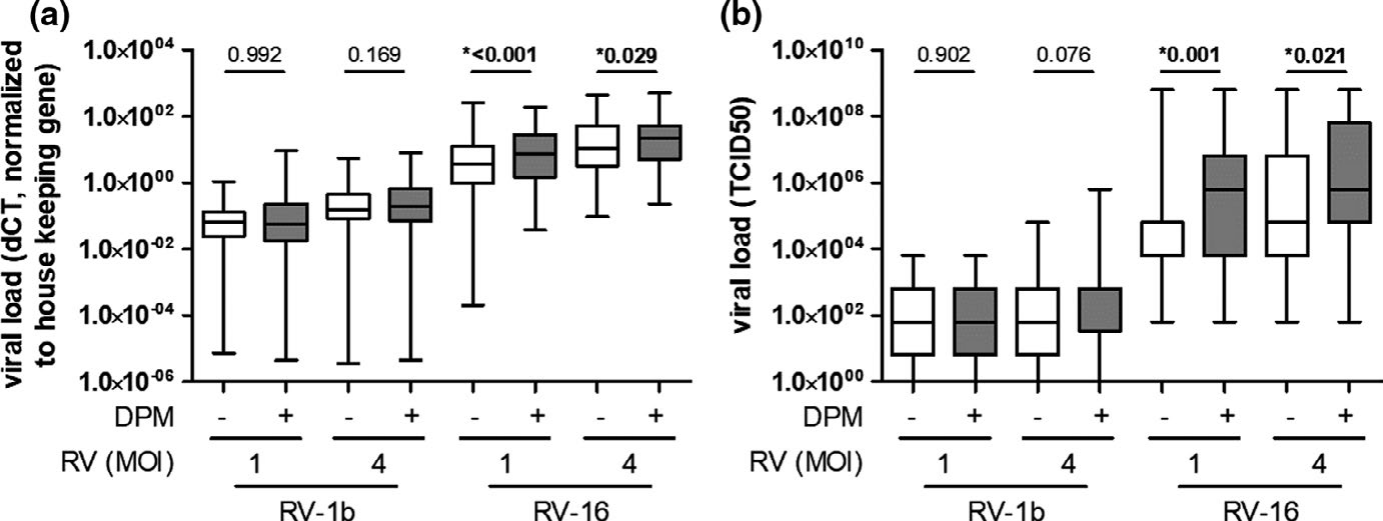
Viral loads of RV‐1b and RV‐16 in nasal epithelial cells (NECs). (a) mRNA levels of viral loads in NECs exposed to diesel particulate matter and non‐exposed controls, measured 20 h post‐infection (*N* = 61). (b) Median tissue culture infection dose (TCID50) in apical washes of NECs exposed to diesel particulate matter and non‐exposed controls, assessed 20 h post‐infection (*N* = 62). Data are shown as a box and whisker plot with line at median, interquartile range (box), and range (line). *p < 0.05, tested with Wilcoxon rank‐sum test

Viral RNA loads and infectious virus release by NECs were significantly higher after RV‐16 infection in NECs exposed to DPM compared with non‐exposed controls (Figure 4). After the RV‐1b infection, the viral loads were lower compared with RV‐16 infection, and there was no effect from previous DPM exposure (Figure 4; Tables S2 and S3). These low viral loads after RV‐1b infection indicate a lower susceptibility of NECs to RV‐1b compared with RV‐16. Therefore, although all RV‐1b data are included in the Supporting Information, we focus on RV‐16 infection in the main manuscript.

### 3.4 DPM exposure affects RV‐16‐induced gene expression of immune factors and cytokine release

**FIGURE 5:**
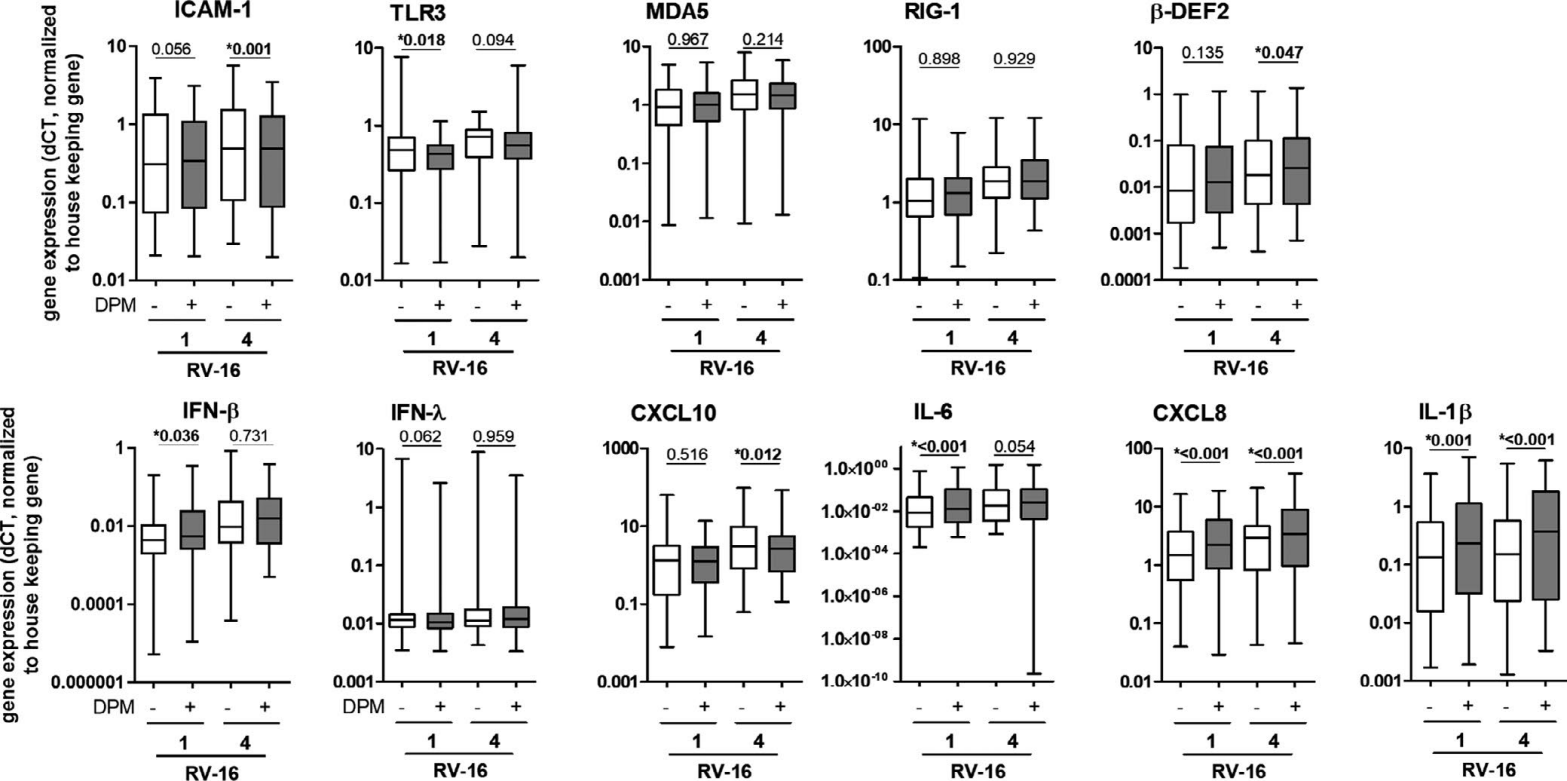
Diesel particulate matter exposure changes the expression of antiviral immune factors in nasal epithelial cells in the context of RV‐16 infection. mRNA levels of different immune factors were assessed by quantitative real‐time RT‐PCR. Data are shown as a box and whiskers plot with line at median, interquartile range (box), and range (line). *N* = 60, *p < 0.05, tested with Wilcoxon rank‐sum test

**FIGURE 6:**
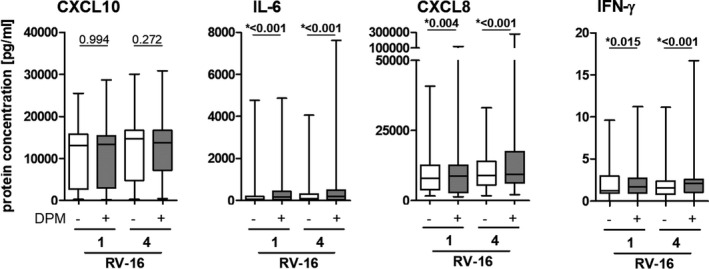
Diesel particulate matter exposure changes protein concentrations in the basolateral media of nasal epithelial cells. Protein levels of CXCL10, IL6, CXCL8, and IFN‐γ were assessed using multiplex bead‐based immunoassays. Data are shown as a box and whiskers plot with line at median, interquartile range (box), and range (line). *N* = 60, *p < 0.05, tested with Wilcoxon rank‐sum test

After finding that DPM exposure increased viral loads, we further explored how previous DPM exposure modifies the antiviral response of NECs to RV‐16 infection. In cells exposed to DPM and infected with RV‐16, expression levels of the RV cell‐surface receptor *ICAM1* were downregulated significantly, but only very modestly. While DPM exposure alone significantly downregulated all PRRs (*TLR3*, *MDA5*, *RIGI*; Figure 2), only *TLR3* was downregulated by DPM exposure when cells were additionally infected with RV‐16 (Figure 5; Table S4). In general, *β*‐*Def2* was only expressed at very low mRNA levels, but was more highly expressed in DPM‐exposed NECs compared with non‐exposed controls upon RV‐16 infection (Figure 5; Table S4). mRNA and protein levels of *IL*‐*6 and CXCL8* and *IL*‐*1β* mRNA were significantly upregulated in cells exposed to DPM upon RV‐16 infection compared with non‐exposed controls (Figure 6; Table S5). For *CXCL10*, we observed a clear downregulation of mRNA levels when exposed to DPM alone (Figure 2), but saw only a small decrease in DPM‐exposed and RV‐16‐infected cells compared with non‐exposed and RV‐16‐infected controls. For the interpretation of this result, we have to consider that RV‐16 infection strongly upregulated *CXCL10* expression (ca. 100‐fold), which may predominate a possible effect of DPM exposure in RV‐16‐infected cells. On the level of CXCL10 protein concentration, we did not find an effect of DPM exposure in the context of RV‐16 infection – probably also because of the strong upregulation due to viral infection. Experiments using RV‐1b instead of RV‐16 showed comparable findings (Table S4).

### 3.5 No differences of the DPM effect between NECs from children and adults

In order to compare the effect of DPM exposure on NECs between children and adults, we normalized the results to the corresponding non‐DPM‐exposed control and then compared normalized, relative values. We did not find any consistent differences in viral titers, viral load, and immune factors between the NECs of children and adults (Tables S6–S8).

## 4 DISCUSSION

DPM exposure of re‐differentiated NECs of children and adults decreased the levels of PRR and CXCL10 and increased the levels of IL6, CXCL8, and *IL1β*. Furthermore, DPM exposure prior to RV‐16 infection resulted in higher viral loads, lower expression of *TLR3* and increased levels of *IFN*‐*β*, *IL1β*, IL6, and CXCL8. Our findings suggest that DPM exposure increased RV‐16 viral loads by downregulation of PPRs and upregulation of pro‐inflammatory cytokines, but not via the cell‐surface receptor ICAM1. Our findings were comparable for NECs of children and adults, indicating no age‐dependent effect of DPM exposure on antiviral response at the level of the nasal epithelium.

Our study shows an enhanced viral load, a decreased antiviral response, and an increase in the (pro‐) inflammatory mediator levels of NECs exposed to DPM prior to RV infection. Our findings are partially in line with the few other studies investigating the effect of air pollution on RV or other respiratory viral infections: Capistrano and colleagues studied the effect of biomass smoke extract exposure on RV‐16 infection in primary human lung fibroblasts and found no effect on viral load and IL6, but higher levels of CXCL8 (Capistrano et al., 2016). Ende and colleagues found that a combination of diesel and house dust mite exposure resulted in impairment of ciliary beating frequency in ALI‐cultured NECs, which was enhanced when the cells were additionally infected with RV. However, the levels of IL6 were not affected by DPM exposure (Ende et al., 2019). An effect of diesel exhaust on influenza infection has also been described. Diesel exposure resulted in an elevated frequency of infected epithelial cells, enhanced IFN‐β levels and enhanced viral attachment due to a higher susceptibility caused by oxidative stress (Jaspers et al., 2005). Further, diesel exposure increased viral load and IFN‐γ levels in human in vivo studies (Noah et al., 2012), and in mice resulted in higher viral titers but not increased IL6 levels (Larcombe et al., 2013). Numerous studies have already shown increased levels of IL6 and CXCL8 due to diesel exhaust or particulate matter exposure in primary respiratory cells (Iwanaga et al., 2013; Kumar et al., 2015; Xian et al., 2020; Zarcone et al., 2016), but not in the context of viral infections as reported in our study. Additionally, reduced CXCL10 levels in ALI‐exposed respiratory epithelial cells after diesel extract exposure (Meldrum et al., 2017) and impairment of cellular integrity after diesel exposure have been reported (Xian et al., 2020; Zarcone et al., 2016; Zhao et al., 2018), which is comparable to our findings. In summary, our findings support the already described effects of diesel exposure in respiratory cells using similar experimental setups. Further, we add novel mechanistic explanations to the research in that field, because, in the context of RV infection, a higher viral load, the impairment of the antiviral response, and the upregulation of the pro‐inflammatory response by exposure to DPM has not yet been reported.

The viral load depends on several factors, including infection dose, virus entry, and viral replication. Since the RV infection dose in our experimental setup was the same for DPM‐exposed and non‐exposed cells, we can exclude this as a reason for the differences found in viral load. However, DPM exposure may increase viral load via the modification of the entrance and replication of the virus. Since we did not find a consistent and biologically relevant effect of DPM exposure on RV cell‐surface receptors (Figures 1 and 4), we think that the DPM effect is not mediated by enhanced viral entrance, but rather by increased viral replication due to impaired host defense mechanisms. However, a study using rat lung epithelial cells found upregulated expression of ICAM‐1 and LDLR after exposure to DPM in submerged conditions, suggesting that DPM exposure may facilitate viral entrance in other settings (Ito et al., 2006). One reason for the difference between the findings could be the timing: the rat study assessed mRNA levels of the binding receptors immediately after a 3 h exposure period, while we chose to wait 20 h after a 4 h exposure period. Future studies should investigate in more detail the impact of the sampling times and thus the timing of the antiviral immune response.

One of the first steps of the antiviral response is the sensing of the virus by PRRs (Stokes et al., 2016). We studied three different PRRs and found for each of them a reduction of mRNA levels after DPM exposure without RV infection. However, after RV‐16 infection following DPM exposure, only TLR3 showed a trend for downregulation. We explain that finding by a stronger stimulatory effect of PRR by RV‐16 infection compared with the DPM‐induced downregulation. Therefore, we hypothesize that the downregulation of PRR could impair the RV sensing and thus inhibit the further cascade of the antiviral response of the cells. However, our findings are in contrary to another study that found an upregulation of TLR3 24 h after a pI:C infection due to a prior 2 h‐exposure to a aqueous‐trapped solution of diesel exhaust (Ciencewicki et al., 2006). These controversial findings indicate that further studies are needed to shed more light on the impact of diesel on viral PPR.

After binding/entrance and sensing by PRRs, the next step of the antiviral response includes antimicrobial peptides, such as defensins. We did not find an effect of DPM exposure on the expression of β‐def2. However, Nam et al. (2006) found an increase of β‐def2 after diesel exposure in IL1β‐primed epithelial cells, indicating that β‐def2 could play a more important role in pre‐damaged epithelia. With the release of IFNs, chemokines, and cytokines, NECs can initiate a peripheral immune response by cross‐activation of other immune cells. DPM exposure prior to RV infection decreased CXCL10 and increased the pro‐inflammatory cytokines *IL1β*, IL6, and CXCL8, and thus modified the antiviral immune response. In line with this, a previous study using bronchial epithelial cells, NECs, and A549 cells also found increased *IL6* in cells exposed to diesel prior to pI:C stimulation (Jaspers et al., 2005). Furthermore, TLR3 priming with pI:C resulted in higher IL6 and CXCL8 levels upon exposure to diesel exhaust particles, suggesting that induction of an antiviral state leads to a more pronounced pro‐inflammatory response after diesel exposure (Bach et al., 2014).

DPM exposure prior to RV infection changed the response of NECs from an antiviral toward a pro‐inflammatory pattern. Pro‐inflammatory mediators, such as IL6, contribute to the antiviral response to clear the invading pathogen. However, an excessive inflammatory response is detrimental to the host. The elevation of various pro‐inflammatory cytokines, as observed in our study, indicate that DPM exposure may cause excessive inflammation, likely damaging the respiratory epithelium. We report a decrease in antiviral chemokines and immune factors, and an excessive increase in pro‐inflammatory cytokines due to DPM exposure. Our findings may explain a potential mechanism impairing the anti‐RV response in NECs, and may—in addition—have an impact on the systemic immune response.

Previous clinical studies in infants (Stern et al., 2013) and adults (Schwartz, 1992) have shown that exposure to air pollution is associated with increased severity and longer duration, rather than increased frequency of respiratory episodes. This indicates that air pollution augments the severity of respiratory complications, which is confirmed by large studies reporting that short‐term air pollution increases asthma attack mortality (Liu et al., 2019) and severity of chronic obstructive pulmonary disease (COPD) exacerbations (Pfeffer et al., 2019). It is further suggested that the majority of asthma and COPD exacerbations are of viral etiology as RVs are detected in up to 80% of exacerbations (Kurai et al., 2013). Our data provide—to the best of our knowledge—for the first‐time, mechanistic evidence for a strong interaction between air pollution and RV infection on the level of the respiratory epithelium, supporting the clinical findings from previous studies.

Our study is the first to investigate the impact of diesel exposure prior to an RV infection using a sophisticated ALI cell model within a large population across an age range from 2.5 months to 65 years. In particular, the pediatric group is the largest so far used to study the effects of diesel exposure on RV infection in vitro. The large group size also enabled the study of age‐dependent effects of air pollution through comparison of the effects of diesel exposure on RV infection in the NECs of children and adults. Even though our cell model closely mimics the nasal mucociliary epithelium, it does not fully model the in vivo situation. The human lungs consist of more than forty different cell types (Ochs & Weibel, 2008), which represent a complex network of interactions. The use of a co‐culture model would at least take into account some of those aspects (Bauer et al., 2015; Ji et al., 2018; Muller et al., 2013). However, it does not reflect the complete in vivo complexity. Another limitation of the study is the acute exposure setting and the high amount of DPM sprayed on the cell cultures. We used a dry powder insufflator, which ensures the deposition of very small particulate agglomerates on the apical cell surface of cell cultures exposed to the air. This better represents the real‐life situation than exposures in submerged settings using suspension of diesel particles. However, the device's handling technique in our study did not enable us to use very low and more realistic doses of DPM. Thus, the DPM dose we used was rather high compared with real‐life exposure. We chose a one‐time exposure of 4 h before the RV infection, thus representing an acute exposure. Although a chronic exposure model would better represent the real‐life scenario, it would require the exposure of the cell cultures for several weeks to low concentrations of air pollution. Such an exposure setting could only be achieved with a much more time‐ and resource‐intensive experimental setup and a different method for DPM exposure. Another limitation of our study is the rather small changes caused by DPM exposure. Most of the significant changes induced by DPM did not reach double or half of the non‐diesel mRNA level, what is usually considered as physiologically relevant. However, most of the significant changes found on protein levels did reach this threshold and therefore could be considered as biologically relevant. The biological relevance of our findings should be further investigated in future studies by measuring more proteins and by including longer time points.

In conclusion, we found that DPM exposure prior to RV infection increases viral loads and affects various immune factors in NECs. This resulted in a weaker antiviral and an excessive inflammatory response following RV infection. Our findings therefore clearly show an impact of diesel exposure on the response of NECs to RV infection. We herewith show mechanistic evidence that air pollution exposure increases the susceptibility of NECs to RV infection. Our findings are relevant for health policy makers and future clinical intervention studies.

## DISCLOSURES

P. Latzin received payment for lectures and/or consultations from Polyphor, Santhera (DMC), Vertex, OM Pharma and Vifor and grants from Vifor and Vertex for projects outside of this study. J. Usemann received payments from Vertex, outside of this study. The two of them and all other authors declare to have no conflicts of interest.

## AUTHOR CONTRIBUTIONS

LM designed the study, performed experiments and analysis, prepared the figures and the main manuscript text. JU performed RT‐PCR measurements, analyzed the data, and wrote the main manuscript text. MPA provided stock of RV‐16 and RV1B and helped with data interpretation. PL developed the project idea and helped with data interpretation. All authors reviewed and approved the manuscript.

## Supporting information



Supplementary Material
