# Supplementary material for: Diesel exposure increases susceptibility of primary human nasal epithelial cells to rhinovirus infection
Source: Physiol Rep. 2021 Sep 20;9(18):e14994. doi: 10.14814/phy2.14994 (PMC8451029; doi:10.14814/phy2.14994)
Supplement: Supplementary file 1 — Supplementary Material [file PHY2-9-e14994-s001.docx]

**Diesel exposure increases susceptibility of primary human nasal epithelial cells to rhinovirus infection**

Loretta Müller^1,2,3,#,^* Jakob Usemann^1,2,3,4#^, Marco P Alves^5,6^, and Philipp Latzin^1,2,3^

^1^Division of Paediatric Respiratory Medicine and Allergology, Department of Paediatrics, Inselspital, Bern University Hospital, University of Bern, Switzerland

^2^Department for BioMedical Research (DBMR), University of Bern, Switzerland

^3^University Children’s Hospital Basel (UKBB), Switzerland

^4^Division of Respiratory Medicine, University Children's Hospital Zurich, Zurich, Switzerland

^5^Institute of Virology and Immunology, Bern, Switzerland

^6^Department of Infectious Diseases and Pathobiology, Vetsuisse Faculty, University of Bern, Bern, Switzerland

^#^These authors contributed equally to this project.

***Corresponding Author:** loretta.mueller@insel.ch; Pediatric Pulmonology, Inselspital Bern, Murtenstrasse 50, 3008 Bern, Switzerland

# Online Supplement (OS)

# Material and Methods

## *Cell culture*

Nasal epithelial cells obtained by nasal brushings (one interdental brush (IDB-G50 3mm, Top Caredent, Zurich, Switzerland) for each nostril, elongated by attaching it to a 200μL pipette tip with parafilm) (passage 0) were seeded in a coated (PureCol Collagen, cat.no. 5005B, Advanced Biomatrix, diluted 1:100 with sterile water, incubated for at least 30min at 37°C) T12.5 tissue culture flask. The cells were cultured until max. 80% confluency was reached (usually reached after 3–4 days) or after a maximum of one week. Then, cells were lifted using Accutase (Sigma-Aldrich, cat.no. A6964-100mL; 8–10min at 37°C, 1mL for a T12.5 tissue culture flask), diluted with 9mL of phosphate-buffered saline (PBS) in a 15mL tube and centrifuged (5min, 300g, room temperature). After aspiration of the supernatant, the cell pellet was resuspended in 1mL PneumaCult Ex Plus media and further propagated in an uncoated T75 tissue culture flask (with 15mL of media in total) until again confluency of max. 80% was reached (usually reached after 5–7 days) or after a maximum of one week. Then the cells were lifted and collected as previously described and seeded as passage 2 in uncoated tissue culture transwells (Corning Transwell polyester membrane inserts, pore size 0.4μm, diameter 12mm, CLS3460-48EA, Sigma-Aldrich) at a density of 100’000 cells/transwell and supplemented with 1mL or 0.5mL PneumaCult Ex Plus in the basolateral and apical chamber, respectively. One day after the cells reached complete confluence (usually 2–5 days after seeding), they were exposed to the air–liquid-interface (ALI) and fed with PneumaCult ALI media from the basolateral chamber only. Normally after about 7–10 days the cell cultures started to produce mucus and after about 14–21 days first motile cilia could be seen. Cell cultures used for the experiments were exposed to the ALI for >28 days.

## *Virus titration*

Viral titers were determined according to the method described in Schögler et al. (1). Briefly, apical washes (50μl final volume) of infected cell cultures were serially diluted in a 96well plate (first row was undiluted) in Dulbecco’s modified Eagle’s medium (Sigma Aldrich, #D5796)containing 4% fetal calf serum (BioConcept, Allschwil, Switzerland). To the 50μl diluted apical wash, we added 50’000 Ohio-HeLa cells (in 150µl media, starting at passage 5) to each of the 96 wells. After 5 days in the incubator (37°C, 5% CO_2_), we counted the infected wells (with destroyed cells) and calculated the 50% tissue culture infective dose (TCID50) per mL of RV using the Spearman-Karber method (2).

## *RNA extraction, cDNA synthesis and quantitative real-time RT-PCR*

Cells were lyzed and fixed using TRizol® (LubioScience, cat.no. 15596018) and stored at -80°C until RNA was isolated with RNA Clean & Concentrator-5 w/ Zymo-Spin IC Columns (Zymo Research) according to the manufacturer’s protocol (including DNAse I treatment). mRNA was converted into cDNA using 200 ng of total RNA with the GoScript Reverse Transcription System (Promega, cat.no. A5003) according to the manufacturer’s protocol). Quantitative real-time RT-PCR was performed using primers and probes for *phosphoglycerate kinase 1* (*PGK1*, housekeeping gene), *RV-16*, *RV-1b, ICAM-1, LDLR, RIG-1, TLR3, MDA5, β-DEF2, IFN-β, IFN-λ, CXCL10, IL-1β, CXCL8* and *IL-6* (sequences presented in Table S1). PCR reactions were carried out using 384-well plates (duplicates, quadruplicates for the housekeeping gene) and contained 5μL master mix (GoTaq qPCR Master Mix system, Promega, cat.no. A6002), 0.5μL forward and reverse primer (10μM), 1μL cDNA and 3μL RNAse-free water. Thermal cycles were run on a Bio-Rad at Biozentrum 7500 Fast Real-Time PCR System (Applied Biosystems/Life Technologies) according to the manufacturer’s protocol. Values were normalized to the housekeeping gene *PGK1* and quantified with the ΔΔCt method.

## *Protein concentrations of cytokine*

Protein concentrations of IFN-γ, CXCL10, CXCL8 and IL-6 were measured in the basolateral media of cell cultures using a human cytokine/chemokine magnetic bead panel (Milliplex MAP kit, Millipore/Merck) and a Magpix Luminex instrument and xponent software (version 4.2, Luminex Corp) according to manufacturer’s instructions. Samples were measured undiluted as single measurements and were incubated overnight at 4°C. We did not analyze RV-1b-infected samples.

# Figures

*
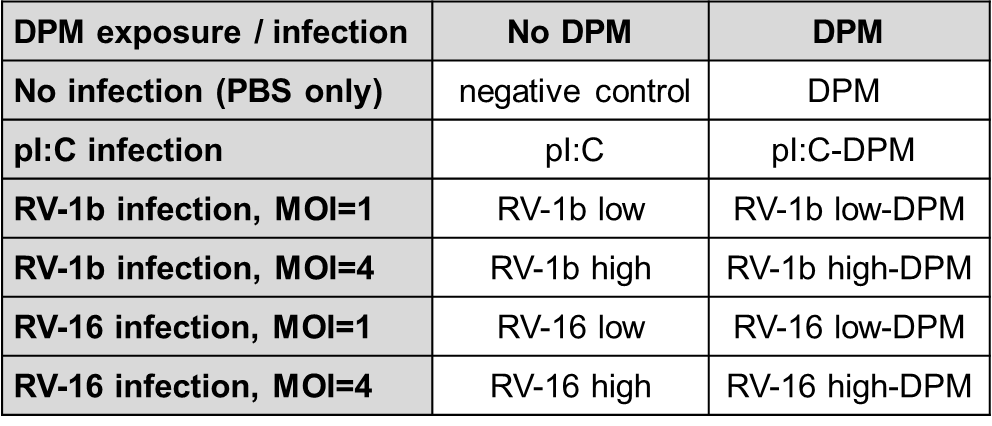
*

***Figure S1. Experimental matrix.*** *Re-differentiated nasal epithelial cells from each participant were used to test twelve different conditions. Six transwells were exposed to diesel particulate matter and six were not exposed and used as non-DPM controls. Two transwells were used as non-infected controls (one with (“DPM”) and one without DPM exposure (“negative control”), Two transwells were infected with pI:C and used positive controls (one with (“pI:C-DPM”) and one without DPM exposure (“pI:C”). Totally, eight transwells were infected with rhinovirus: four with RV-1b and four with RV-16 (two each with the low and two with the high dose) and half of them were also exposed to DPM. DPM: diesel particulate matter; MOI:* multiplicity of infection.

***
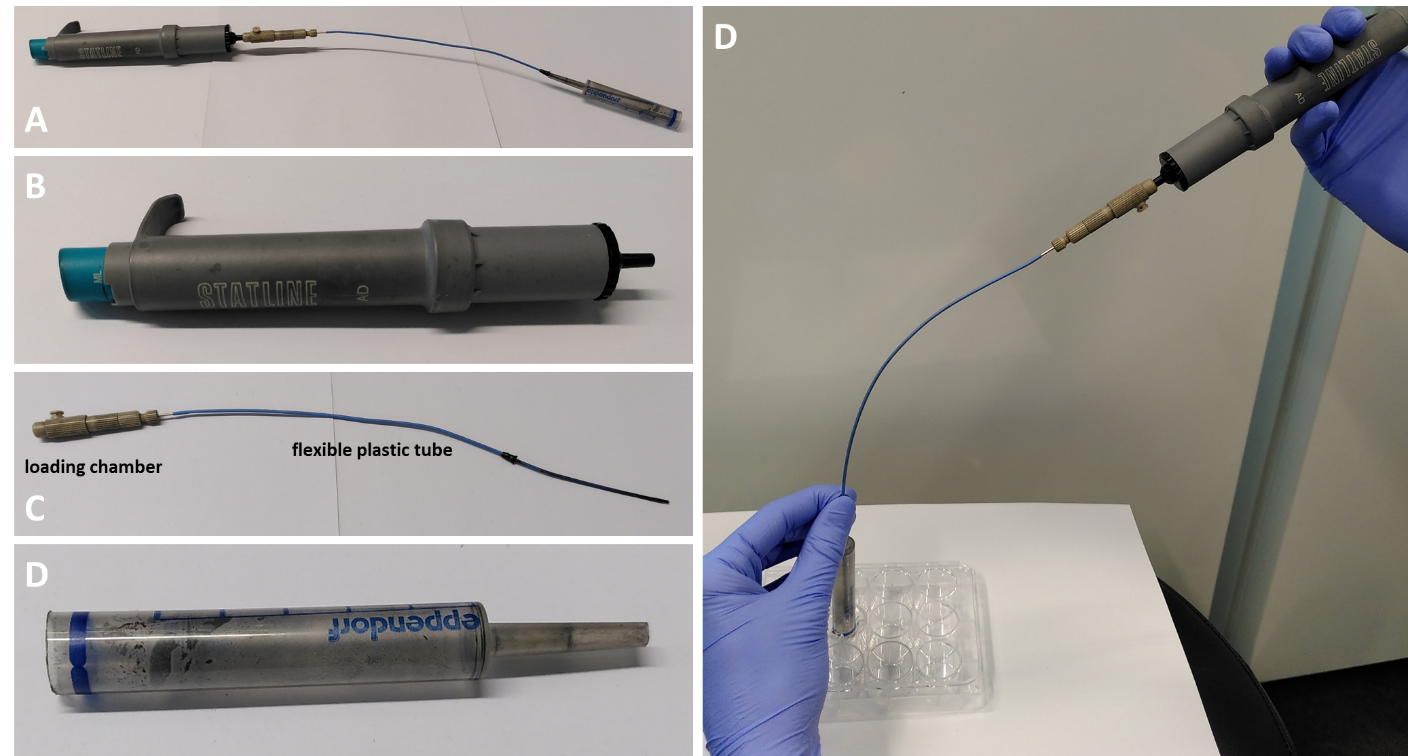
***

***Figure S2. Dry Powder Insufflator used for the exposure to diesel particulate matter (DPM). (A)*** *The whole device consisting of the air pump model AP-1* ***(B)****, a custom-made extra-long and flexible plastic tube attached to the loading chamber (model DP-4-CPL)* ***(C)****. We prepared a 5mL Eppendorf combitip to be used as a distance holder and exposure chamber to avoid DPM flying to other transwells. (D) DPM was loaded into the loading chamber, the air pump was attached, the tube inserted into the Eppendorf combitip and this was placed on the desired transwell. Then one puff of the air pump (set to 200μL) was applied and the DPM was deposited on the transwells.*

***
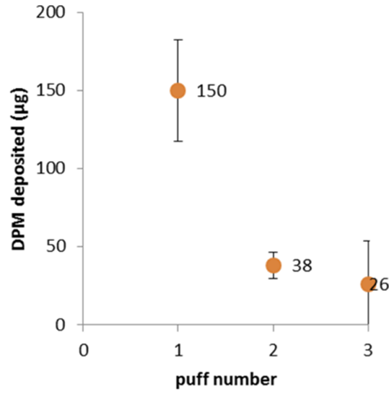
***

***Figure S3. Characterization of the spray pattern of the Dry Powder Insufflator.*** *300µg DPM were loaded into the insufflator and the quantities of DPM released by the first, the second and the third puff (of 200μL each) were identified. N=3.*

**

***Figure S4. Toxicity of DPM exposure measured by the release of lactate dehydrogenase.*** *DPM exposure did not increase the release of lactate dehydrogenase (assessed using the lactate dehydrogenase (LDH) cytotoxicity assay kit from Pierce, ordered from LubioScience, samples run in duplicates) in apical washes of nasel epithelial cells (NECs) 24h after start of the DPM exposure.* *N=2.*

# Tables

***Table S1. Primer Sequences for quantitative real-time RT-PCR.***

| **Target** | **forward primer** | **reverse primer** | **NCBI reference** |
| --- | --- | --- | --- |
| *PGK1* | GACCGAATCACCGACCTCTC | GTCGACTCTCATAACGACCC | [NM_000291.3](https://www.ncbi.nlm.nih.gov/nucleotide/183603937?report=genbank&log$=nucltop&blast_rank=1&RID=C2AKE7RS013) |
| *RV-16* | GGTTGGTCGCTCAGCTGTTA | CGGCTCTTCACACCTTGTCC | HQ336416.1 |
| *RV-1B* | TGACCTGCCCGATAAGGGTA | TGGTGGGTGGAGCTAGAAGA | KC881035.1 |
| *ICAM-1* | CTTCGTGTCCTGTATGGCCC | CTGGCACATTGGAGTCTGCT | NM_000201.2 |
| *LDLR* | CTCAGTTCTGGAGGTGCGAT | AGACGAACTGCCGAGAGATG | NM_000527.4 |
| *RIG-1* | GCCTTCAGACATGGGACGAA | ACTGCTTTGGCTTGGGATGT | NM_014314.3 |
| *TLR3* | GGACTTTGAGGCGGGTGTTT | TCGCAAACAGAGTGCATGGT | NM_003265.2 |
| *MDA5* | AGCTTCACCTGGTGTTGGAG | TGGCAAACTTCTTGCATGGC | NM_022168.3 |
| *β defensin 2* | GCCATGAGGGTCTTGTATCTCC | AAGGCAGGTAACAGGATCGC | NM_001205266.1 |
| *IFN-β* | ACGCCGCATTGACCATCTAT | TGGCCTTCAGGTAATGCAGA | [NM_002176.3](https://www.ncbi.nlm.nih.gov/nucleotide/930588924?report=genbank&log$=nucltop&blast_rank=1&RID=C2APYZVH013) |
| *IFN-λ* | CCAGAATGTGACGCTGCTCTC | GCACACTCTTCCACTTCGC | NM_170743.3 |
| *CXCL10* | CTAGAACTGTACGCTGTACC | CTTGATGGCCTTCGATTCTG | NM_001565.3 |
| *IL-1β* | TCGCCAGTGAAATGATGGCT | TGGAAGGAGCACTTCATCTGTT | NM_000576.2 |
| *CXCL8* | CTGATTTCTGCAGCTCTGTG | ATTTCTGTGTTGGCGCAGTG | NM_000584.3 |
| *IL-6* | TACATCCTCGACGGCATCTC | GCCATCTTTGGAAGGTTCAG | [NM_000600.4](https://www.ncbi.nlm.nih.gov/nucleotide/969812508?report=genbank&log$=nucltop&blast_rank=4&RID=C2ANSCU6013) |

***Table S2. Effect of DPM exposure on viral load levels in NECs of children and adults (RT-PCR).*** *Data are presented as median (range) of the mRNA levels normalized to the house keeping gene PGK1 (ΔCt).* *Total N=60, children N=48, adults N=12. *p<0.05, tested with Wilcoxon rank-sum test.*

|  | **All** | | | **Children** | | | **adults** | | |
| --- | --- | --- | --- | --- | --- | --- | --- | --- | --- |
| **Viral load** | **no diesel** | **diesel** | **p-value** | **no diesel** | **diesel** | **p-value** | **no diesel** | **diesel** | **p-value** |
| **RV1b**  **low** | 0.066 (0.001−1.096) | 0.055  (0.001− 9.188) | 0.992 | 0.068 (0.005−1.096) | 0.061  (0.006−9.188) | 0.956 | 0.001 (0.001−.493) | 0.002 (0.001−0.258) | 0.594 |
| **RV1b**  **high** | 0.149 (0.001−5.536) | 0.203 (0.001−7.876) | 0.169 | 0.173  (0.361−5.536) | 0.234  (0.039−7.876) | 0.319 | 0.001  (0.001−2.173) | 0.001 (0.001−5.37649) | 0.208 |
| **RV16**  **Low** | 3.709 (0.001−259.259) | 7.028  (0.037− 189.263) | **<0.001** | 5.580  (0.001−259.259) | 10.746  (0.037−189.263) | **<0.001** | 1.075  (0.083−7.786) | 1.609  (0.148−6.451) | 0.424 |
| **RV16**  **high** | 10.078 (0.093−453.906) | 21.388 (0.0233−514.757) | **0.029** | 17.315  (0.09−453.906) | 31.779  (0.232−514.757) | 0.108 | 2.956 (0.346−10.078) | 3.816  (0..869−36.186) | **0.041** |

***Table S3. Effect of DPM exposure on viral load levels in NECs of children and adults (TCID50).*** *Data are presented as median (range) of the TCID50.* *Total N=61, children N=49, adults N=12. *p<0.05, tested with Wilcoxon rank-sum test.*

|  | **All** | | | **Children** | | | **adults** | | |
| --- | --- | --- | --- | --- | --- | --- | --- | --- | --- |
|  | **no diesel** | **diesel** | **p-value** | **no diesel** | **diesel** | **p-value** | **no diesel** | **diesel** | **p-value** |
| **control** | 6.32  (6.32−63.2) | 6.32  (6.32−632) | 0.317 | 6.32  (6.32−63.2) | 6.32  (6.32−632) | 0.317 | 6.32  (6.32−63.2) | 6.32  (6.32−63.2) | n/a |
| **pI:C** | 6.32  (0.0−63.2) | 6.32  (0.0−632) | 0.157 | 6.32  (0.0−63.2) | 6.32  (0.0−632) | 0.157 | 6.32 (6.32−63.2) | 6.32  (6.32−63.2) | n/a |
| **RV1b**  **low** | 63.2  (0.0−6320) | 6.32 (0.0−6320) | 0.902 | 63.2  (0.0−6320) | 63.2 (0.0−6320) | 0.369 | 63.2 (6.32−6320) | 63.2  (6.32−632) | 0.077 |
| **RV1b**  **high** | 63.2  (0.0−63200) | 632 (0.0−632000) | 0.076 | 63.2  (0.0−63200) | 632 (0.0−632000) | **0.033** | 632  (6.32−6320) | 632  (63.2−632) | 0.583 |
| **RV16**  **Low** | 6320  (63.2−63.32^+08^) | 632000  (63.2−63.32^+08^) | **0.001** | 6320  (63.2−63.32^+08^) | 632000  (63.2−63.32^+08^) | **0.003** | 6320  (63.2−63.32^+08^) | 63200  (63.2−63.32^+07^) | 0.202 |
| **RV16**  **high** | 63200  (63.2−63.32^+08^) | 632000  (63.2−63.32^+08^) | **0.021** | 632000  (63.2−63.32^+08^) | 632000  (63.2−63.32^+08^) | **0.038** | 6320  (63.2−63.32^+07^) | 63200 (63.2−63.32^+08^) | 0.119 |

***Table S4 (part 1). Effect of DPM exposure on mRNA levels of various immune factors (RT-PCR).*** *Data are presented as median (range) of the mRNA levels. Total N=60, children N=48, adults N=12. *p<0.05, tested with Wilcoxon rank-sum test.*

|  | **All** | | | **Children** | | | **adults** | | |
| --- | --- | --- | --- | --- | --- | --- | --- | --- | --- |
| **ICAM-1** | **no diesel** | **diesel** | **p-value** | **no diesel** | **diesel** | **p-value** | **no diesel** | **diesel** | **p-value** |
| **control** | 0.238  (0.018−4.021) | 0.244  (0.001−4.288) | **0.043** | 0.169  (0.01−3.195) | 0.134  (0.017−2.409) | 0.143 | 1.352 (0.044−4.021) | 1.223 (0.058−4.288) | 0.158 |
| **pI:C** | 0.341  (0.022−5.843) | 0.330  (0.010−5.254) | 0.834 | 0.279  (0.022−5.843) | 0.255  (0.020−5.254) | 0.996 | 2.050 (0.115−3.374) | 1.916  (0.163−4.917) | 0.859 |
| **RV1b**  **low** | 0.225  (0.022−3.640) | 0.196  (0.001−2.158) | 0.107 | 0.191  (0.022−3.640) | 0.129  (0.018−2.158) | 0.512 | 1.031 (0.122−1.821) | 1.037  (0.098−1.213) | **0.028** |
| **RV1b**  **high** | 0.303  (0.022−5.964) | 0.247  (0.001−4.917) | **0.010** | 0.214  (0.022−5.964) | 0.211  (0.016−2.546) | **0.005** | 1.004 (0.106−1.911) | 1.034 (0.141−4.917) | 0.678 |
| **RV16**  **Low** | 0.309  (0.020−3.917) | 0.338  (0.001−3.116) | 0.056 | 0.179  (0.020−3.917) | 0.243  (0.020−3.116) | 0.179 | 1.405  (0.169−2.713) | 1.328  (0.144−2.412) | 0.182 |
| **RV16**  **high** | 0.487  (0.029−5.700) | 0.485  (0.001−3.454) | **0.001** | 0.301  (0.029−4.536) | 0.353  (0.019−3.454) | 0.057 | 1.483 (0.152−5.700) | 1.338  (0.147−3.271) | **0.005** |
| **LDLR** | **no diesel** | **diesel** | **p-value** | **no diesel** | **diesel** | **p-value** | **no diesel** | **diesel** | **p-value** |
| **control** | 0.178 (0.038−1.363) | 0.177  (0.038−1.072) | 0.117 | 0.169  (0.038−0.865) | 0.155 (0.038−1.072) | 0.144 | 0.528  (0.066−1.363) | 0.634 (0.066−0.876) | 0.787 |
| **pI:C** | 0.218  (0.034−1.286) | 0.241  (0.045−1.757) | **0.002** | 0.279  (0.034−1.286) | 0.183 (0.045−1.271) | **0.016** | 0.629  (0.218−1.178) | 1.060  (0.570−1.757) | 0.049 |
| **RV1b**  **low** | 0.154 (0.031−0.944) | 0.177  (0.039−0.950) | **<0.001** | 0.191  (0.031−0.944) | 0.145 (0.039−0.950) | **<0.001** | 0.418 (0.095−0.790) | 0.535 (0.117−0.741) | 0.327 |
| **RV1b**  **high** | 0.166  (0.041−0.866) | 0.183  (0.043−1.458) | **<0.001** | 0.214  (0.041−0.866) | 0.173 (0.043−1.285) | **<0.001** | 0.399 (0.120−0.668) | 0.581 (0.129−1.458) | 0.161 |
| **RV16**  **Low** | 0.183 (0.015−1.236) | 0.260  (0.046−1.603) | **<0.001** | 0.179  (0.015−1.236) | 0.221 (0.046−1.456) | **<0.001** | 0.639 (0.243−1.069) | 0.678 (0.259−1.603) | 0.059 |
| **RV16**  **high** | 0.250  (0.048−1.572) | 0.294 (0.069−1.489) | **<0.001** | 0.301  (0.048−1.322) | 0.238 (0.069−1.420) | **0.007** | 0.747 (0.263−1.572) | 0.916  (0.605−1.489) | 0.213 |
| **TLR3** | **no diesel** | **diesel** | **p-value** | **no diesel** | **diesel** | **p-value** | **no diesel** | **diesel** | **p-value** |
| **control** | 0.270  (0.007−1.435) | 0.217  (0.005−1.175) | **<0.001** | 0.270 (0.130−1.369) | 0.226  (0.099−0.796) | **<0.001** | 0.268  (0.007−1.435) | 0.138  (0.005−1.175) | 0.158 |
| **pI:C** | 0.374 (0.007−1.502) | 0.405 (0.019−3.226) | **0.043** | 0.372 (0.124−1.255) | 0.366 (0.118−3.226) | **0.003** | 0.555  (0.007−1.502) | 0.679 (0.019−1.312) | 0.859 |
| **RV1b**  **low** | 0.280  (0.006−1.261) | 0.227 (0.005−1.493) | **0.001** | 0.281  (0.1421−1.261) | 0.237 (0.095−1.493) | 0.161 | 0.273 (0.006−0.555) | 0.120 (0.005−0.477) | **0.021** |
| **RV1b**  **high** | 0.316  (0.014−0.688) | 0.233 (0.006−0.679) | **<0.001** | 0.316 (0.163−0.688) | 0.238 (0.153−0.508) | **<0.001** | 0.346 (0.014−0.645) | 0.227  (0.006−0.679) | 0.066 |
| **RV16**  **Low** | 0.484  (0.016−7.674) | 0.425  (0.017−1.134) | **0.018** | 0.487 (0.204−7.674) | 0.446 (0.145−1.134) | 0.095 | 0.484 (0.016−4.752) | 0.267  (0.017−0.785) | **0.050** |
| **RV16**  **high** | 0.717 (0.027−1.512) | 0.550 (0.019−5.986) | 0.094 | 0.652 (0.190−1.423) | 0.554 (0.221−5.986) | 0.351 | 0.727  (0.027−1.512) | 0.523 (0.019−1.665) | **0.049** |
| **MDA5** | **no diesel** | **diesel** | **p-value** | **no diesel** | **diesel** | **p-value** | **no diesel** | **diesel** | **p-value** |
| **control** | 0.280  (0.016−2.995) | 0.251 (0.010−5.043) | **<0.001** | 0.291 (0.083−2.99) | 0.258 (0.082−2.016) | **<0.001** | 0.147  (0.016−2.707) | 0.051  (0.010−5.043) | 0.182 |
| **pI:C** | 0.893 (0.016−7.594) | 0.642 (0.016−6.73) | 0.913 | 0.728  (0.086−7.59) | 0.689 (0.091−5.721) | 0.634 | 0.997 (0.016−3.910) | 0.570  (0.016−6.730) | 0.441 |
| **RV1b**  **low** | 0.308 (0.049−2.069) | 0.297  (0.057−1.471) | **0.001** | 0.313  (0.072−2.069) | 0.315 (0.084−1.471) | **0.002** | 0.255 (0.049−1.420) | 0.238 (0.057−0.835) | 0.038 |
| **RV1b**  **high** | 0.381  (0.065−2.493) | 0.324 (0.056−2.894) | **<0.001** | 0.370  (0.101−2.493) | 0.326 (0.103−2.126) | **0.004** | 0.4766  (0.065−1.919) | 0.189  (0.056−2.894) | 0.109 |
| **RV16**  **Low** | 0.922  (0.008−4.865) | 1.010 (0.011−5.385) | 0.967 | 1.038  (0.182−4.86) | 1.131 (0.147−5.385) | 0.652 | 0.451  (0.008−3.611) | 0.247  (0.011−2.543) | 0.286 |
| **RV16**  **high** | 1.523  (0.009−7.947) | 1.450  (0.012−5.860) | 0.214 | 1.630 (0.346−7.947) | 1.591  (0.396−5.860) | 0.498 | 0.613  (0.009−5.975) | 0.424  (0.012−2.528) | 0.158 |

***Table S4 (part 2). Effect of DPM exposure on mRNA levels of various immune factors (RT-PCR).*** *Data are presented as median (range) of the mRNA levels. Total N=60, children N=48, adults N=12. *p<0.05, tested with rank-sum Wilcoxon test.*

|  | **All** | | | **Children** | | | **adults** | | |
| --- | --- | --- | --- | --- | --- | --- | --- | --- | --- |
| **RIG-1** | **no diesel** | **diesel** | **p-value** | **no diesel** | **diesel** | **p-value** | **no diesel** | **diesel** | **p-value** |
| **control** | 0.266  (0.046−4.038) | 0.193  (0.023−3.829) | **<0.001** | 0.291  (0.079−4.038) | 0.258  (0.087−1.807) | 0.099 | 0.147  (0.046−1.387) | 0.051  (0.023−3.829) | **0.001** |
| **pI:C** | 1.035 (0.021−8.970) | 0.897 (0.086−6.937) | 0.357 | 0.728  (0.072−8.970) | 0.689 (0.086−6.937) | 0.302 | 0.997  (0.021−3.937) | 0.570 (0.150−4.225) | 0.953 |
| **RV1b**  **low** | 0.281 (0.021−2.720) | 0.211 (0.027−1.749) | **<0.001** | 0.313  (0.088−2.720) | 0.315 (0.099−1.749) | **0.001** | 0.255 (0.021−0.667) | 0.238 (0.027−0.456) | **0.015** |
| **RV1b**  **high** | 0.412  (0.032−3.447) | 0.315 (0.023−3.266) | **<0.001** | 0.370  (0.101−3.447) | 0.326 (0.102−3.266) | **0.001** | 0.476  (0.032−0.982) | 0.189  (0.023−2.158) | 0.109 |
| **RV16**  **Low** | 1.048 (0.106−11.689) | 1.304 (0.149−7.859) | 0.898 | 1.038  (0.106−6.326) | 1.131  (0.206−7.859) | 0.454 | 0.451  (0.193−11.689) | 0.247  (0.149−3.429) | 0.091 |
| **RV16**  **high** | 1.858  (0.222−12.104) | 1.842  (0.433−12.132) | 0.929 | 1.630 (0.222−12.104) | 1.591  (0.636−12.132) | 0.328 | 0.613 (0.312−7.565) | 0.424  (0.433−4.761) | 0.099 |
| **βDEF2** | **no diesel** | **diesel** | **p-value** | **no diesel** | **diesel** | **p-value** | **no diesel** | **diesel** | **p-value** |
| **control** | 0.005 (0.001−1.230) | 0.008 (0.001−0.797) | 0.551 | 0.002 (0.001−0.883) | 0.003 (0.001−0.559) | 0.211 | 0.067 (0.001−1.230) | 0.056 (0.001−0.797) | 0.272 |
| **pI:C** | 0.030  (0.001−1.62) | 0.033  (0.001−2.082) | **0.020** | 0.013 (0.001−0.593) | 0.016 (0.001−1.375) | **0.016** | 0.080  (0.007−1.622) | 0.144 (0.022−2.082) | 0.594 |
| **RV1b**  **low** | 0.005 (0.001−0.557) | 0.007 (0.001−0.453) | **0.032** | 0.002 (0.001−0.244) | 0.003 (0.001−0.368) | **0.006** | 0.058 (0.001−0.557) | 0.063 (0.003−0.453) | 0.374 |
| **RV1b**  **high** | 0.006  (0.001−0.585) | 0.009 (0.001−0.584) | 0.097 | 0.002 (0.001−0.335) | 0.004 (0.001−0.376) | **0.025** | 0.054  (0.002−0.585) | 0.063 (0.001−0.584) | 0.678 |
| **RV16**  **Low** | 0.008  (0.001−0.986) | 0.013 (0.001−1.182) | 0.135 | 0.0047  (0.001−0.407) | 0.005 (0.001−0.290) | **0.049** | 0.074 (0.006−0.986) | 0.071 (0.002−1.182) | 0.594 |
| **RV16**  **high** | 0.018 (0.001−1.174) | 0.026 (0.001−1.375) | **0.047** | 0.010  (0.001−0.468) | 0.015 (0.001−1.375) | **0.010** | 0.155  (0.009−1.174) | 0.051 (0.019−1.074) | 0.480 |
| **IFN-β** | **no diesel** | **diesel** | **p-value** | **no diesel** | **diesel** | **p-value** | **no diesel** | **diesel** | **p-value** |
| **control** | 0.001 (0.001−0.100) | 0.001 (0.001−0.020) | 0.523 | 0.001 (0.001−0.100) | 0.001  (0.001−0.020) | 0.959 | 0.001 (0.001−0.004) | 0.008 (0.001−0.005) | 0.207 |
| **pI:C** | 0.001 (0.001−0.031) | 0.001 (0.001−0.045) | 0.629 | 0.001 (0.001−0.031) | 0.001 (0.001−0.045) | 0.780 | 0.001 (0.001−0.004) | 0.001 (0.001−0.010) | 0.575 |
| **RV1b**  **low** | 0.001 (0.001−0.084) | 0.001 (0.001−0.024) | 0.892 | 0.001 (0.001−0.084) | 0.001 (0.001−0.024) | 0.889 | 0.001 (0.001−0.004) | 0.001 (0.001−0.001) | 0.655 |
| **RV1b**  **high** | 0.001 (0.001−0.026) | 0.001 (0.001−0.065) | 0.069 | 0.001  (0.001−0.026) | 0.001  (0.001−0.065) | 0.983 | 0.001  (0.001− 0.008) | 0.001 (0.001−0.010) | 0.225 |
| **RV16**  **Low** | 0.004 (0.001−0.201) | 0.005 (0.001−0.353) | **0.036** | 0.004  (0.001−0.201) | 0.005 (0.001−0.353) | **0.039** | 0.004  (0.001−0.054) | 0.004 (0.001−0.060) | 0.859 |
| **RV16**  **high** | 0.009 (0.001−0.855) | 0.016 (0.001−0.387) | 0.731 | 0.011 (0.001−0.855) | 0.015 (0.001−0.387) | 0.523 | 0.007  (0.001−0.197) | 0.031  (0.001−0.116) | 0.515 |
| **IFN-λ** | **no diesel** | **diesel** | **p-value** | **no diesel** | **diesel** | **p-value** | **no diesel** | **diesel** | **p-value** |
| **control** | 0.010  (0.002−2.152) | 0.009 (0.002−1.068) | 0.057 | 0.001 (0.003−0.027) | 0.001  (0.004−0.041) | 0.150 | 0.013 (0.002−2.152) | 0.014 (0.002−1.068) | 0.209 |
| **pI:C** | 0.009  (0.004−3.042) | 0.010  (0.003−3.08) | 0.121 | 0.001 (0.004−0.055) | 0.001  (0.005−0.072) | 0.227 | 0.015 (0.004−3.042) | 0.025  (0.003−3.085) | 0.374 |
| **RV1b**  **low** | 0.010 (0.003−0.039) | 0.010 (0.003−0.035) | 0.245 | 0.001 (0.005−0.039) | 0.001  (0.004−0.035) | 0.797 | 0.0147 (0.003−0.023) | 0.010 (0.003−0.025) | 0.109 |
| **RV1b**  **high** | 0.009 (0.003−0.036) | 0.010  (0.002−0.051) | 0.943 | 0.001  (0.005−0.034) | 0.001  (0.004−0.038) | 0.974 | 0.011  (0.003−0.036) | 0.012 (0.002−0.051) | 0.953 |
| **RV16**  **Low** | 0.011 (0.003−6.852) | 0.010  (0.003−2.591) | 0.062 | 0.004  (0.003−0.043) | 0.005 (0.004−0.038) | 0.094 | 0.019 (0.003−6.852) | 0.019  (0.003−2.591) | 0.328 |
| **RV16**  **high** | 0.011 (0.004−8.858) | 0.011 (0.003−3.540) | 0.959 | 0.011 (0.004−0.053) | 0.015 (0.005−0.049) | 0.642 | 0.026  (0.004−8.858) | 0.032 (0.003−3.540) | 0.346 |

***Table S4 (part 3). Effect of DPM exposure on mRNA levels of various immune factors (RT-PCR).*** *Data are presented as median (range) of the mRNA levels. Total N=60, children N=48, adults N=12. *p<0.05, tested with Wilcoxon rank-sum test.*

|  | **All** | | | **Children** | | | **adults** | | |
| --- | --- | --- | --- | --- | --- | --- | --- | --- | --- |
| **CXCL10** | **no diesel** | **diesel** | **p-value** | **no diesel** | **diesel** | **p-value** | **no diesel** | **diesel** | **p-value** |
| **control** | 0.040  (0.001−3.523) | 0.018 (0.001−15.47) | **0.001** | 0.024  (0.001− 3.057) | 0.001  (0.001− 2647) | **0.001** | 0 .249  (0.006−3.523) | 0.082  (0.004−15.472) | 0.059 |
| **pI:C** | 1.545  (0.001−46.316) | 0.774  (0.003−32.022) | 0.416 | 0.355  (0.001−35.512) | 0.543  (0.003−32.022) | 0.367 | 5.00  (0.176−46.316) | 3.803  (0.273−29.161) | 0.767 |
| **RV1b**  **low** | 0.091 (.00262−4.030) | 0.041 (0.001−1.598) | **0.001** | 0.029 (0.003−3.510) | 0.027  (0.001−0.918) | **0.038** | 0.363  (0.007−4.030) | 0.146  (0.008−1.598) | **0.011** |
| **RV1b**  **high** | 0.090  (0.001− 6.717) | 0.077  (0.002−4.095) | **0.001** | 0.068  (0.001−5.101) | 0.063  (0.002−2.031) | **0.001** | 1.866  (0.014−6.717) | 0.316  (0.009−4.095) | 0.085 |
| **RV16**  **Low** | 1.293 (0.007−63.140) | 1.264  (0.014−13.851) | 0.516 | 1.177  (0.008−63.140) | 1.119  (0.015−13.851) | 0.976 | 3.116  (0.148−22.343) | 1.310  (0.333−9.540) | 0.182 |
| **RV16**  **high** | 3.101 (0.061−97.252) | 2.685 (0.116−84.492) | **0.012** | 2.575  (0.061−37.116) | 2.335  (0.116−84.492) | 0.119 | 10.595  (0.564−97.252) | 3.808  (0.159−15.142) | **0.023** |
| **IL1-β** | **no diesel** | **diesel** | **p-value** | **no diesel** | **diesel** | **p-value** | **no diesel** | **diesel** | **p-value** |
| **control** | 0.132  (0.001−3.27) | 0.218  (0.003−5.172) | **0.001** | 0.083  (0.001−3.271) | 0.066  (0.003− 5.172) | **<0.001** | 0.793  (0.120−1.845) | 1.475  (0.072−4.504) | **0.008** |
| **pI:C** | 0.125  (0.002−2.630) | 0.235  (0.003−8.669) | **<0.001** | 0.058  (0.002−2.630) | 0.101  (0.003−8.669) | **<0.001** | 0.654  (0.136−1.668) | 1.177  (0.299−1.964) | **0.012** |
| **RV1b**  **low** | 0.106  (0.004−3.605) | 0.191  (0.007−6.409) | **<0.001** | 0.063  (0.004−3.605) | 0.115  (0.007−6.409) | **<0.001** | 0.462  (0.162−2.060) | 0.971  (0.122−2.274) | 0.263 |
| **RV1b**  **high** | 0.120  (0.004−3.232) | 0.228  (0.008−6.189) | **<0.001** | 0.069  (0.001−3.232) | 0.201  (0.008−6.189) | **<0.001** | 0.434  (0.139−1.472) | 1.051  (0.138−2.330) | **0.017** |
| **RV16**  **Low** | 0.135  (0.002− 3.623) | 0.235  (0.001−7.080) | **<0.001** | 0.083  (0.003−3.623) | 0.110  (0.001−7.080) | **<0.001** | 0.408  (0.087−1.636) | 1.008  (0.047−3.062) | **0.009** |
| **RV16**  **high** | 0.151 (0.001−5.432) | 0.375  (0.003−6.183) | **<0.001** | 0.109  (0.001−3.332) | 0.166 (0.003−6.183) | **<0.001** | 0.467  (0.126−5.432) | 1.165  (0.128−4.607) | **0.026** |
| **IL-6** | **no diesel** | **diesel** | **p-value** | **no diesel** | **diesel** | **p-value** | **no diesel** | **diesel** | **p-value** |
| **control** | 0.006  (0.001−0.396) | 0.06  (0.001−0.567) | **<0.001** | 0.002  (0.001−0.117) | 0.002  (0.001−0.333) | **0.005** | 0.098 (0.001−0.396) | 0.134  (0.001−0.567) | 0.186 |
| **pI:C** | 0.007  (0.001−1.030) | 0.014  (0.001−1.595) | **<0.001** | 0.005 (0.001−1.030) | 0.005  (0.001− 0.841) | **0.004** | 0.132 (0.006−0.848) | 0.303  (0.018−1.595) | **0.021** |
| **RV1b**  **low** | 0.003  (0.001−0.486) | 0.006  (0.001−0.553) | **<0.001** | 0.002  (0.001−0.337) | 0.004  (0.001−0.553) | **0.001** | .0297 (0.003−0.486) | 0.048  (0.002−0.277) | 0.594 |
| **RV1b**  **high** | 0.004  (0.001−0.386) | 0.006  (0.001−0.449) | **<0.001** | 0.003  (0.001−0.301) | 0.004  (0.001− 0.296) | **0.003** | 0.033 (0.002−0.386) | 0.252  (0.002−0.490) | **0.021** |
| **RV16**  **Low** | 0.008  (0.001−0.749) | 0.012  (0.001−1.124) | **<0.001** | 0.004  (0.001−0.316) | 0.008 (0.001−0.490) | **<0.001** | 0.038 (0.004−0.749) | 0.132 (0.002−1.124) | **0.007** |
| **RV16**  **high** | 0.018 (0.001−1.470) | 0.025  (0.001−1.508) | 0.054 | 0.011 (0.001−0.681) | 0.013 (0.001−0.903) | 0.392 | 0.101  (0.006−1.470) | 0.214 (0.011−1.508) | 0.084 |
| **CXCL8** | **no diesel** | **diesel** | **p-value** | **no diesel** | **diesel** | **p-value** | **no diesel** | **diesel** | **p-value** |
| **control** | 0.980 (0.009−21.035) | 1.227  (0.009−18.645) | **0.052** | 0.980  (0.185−21.035) | 1.227  (0.284−18.645) | **<0.001** | 0.846 (0.009−8.701) | 1.229  (0.009−12.312) | 0.136 |
| **pI:C** | 1.529  (0.023−32.780) | 2.152 (0.049−36.975) | **<0.001** | 1.659 (0.168−32.780) | 2.228 (0.187−36.975) | **<0.001** | 0.958 (0.023−5.730) | 1.121  (0.049−11.570) | 0.155 |
| **RV1b**  **low** | 1.062 (0.021−18.964) | 1.234  (0.026−29.466) | **0.001** | 1.178  (0.274−18.964) | 1.414 (0.343−29.466) | **<0.001** | 0.315 (0.021−5.883) | 0.397 (0.026−3.103) | 0.136 |
| **RV1b**  **high** | 0.891 (0.021−23.539) | 1.476  (0.014−16.839) | **<0.001** | 0.974 (0.091−23.539) | 1.728 (0.340−16.839) | **0.001** | 0.358  (0.021− 3.540) | 0.377 (0.014−11.137) | 0.859 |
| **RV16**  **Low** | 1.467  (0.039−16.427) | 2.208 (0.028−18.970) | **<0.001** | 1.920 (0.286−16.427) | 2.453 (0.417−16.293) | **<0.001** | 0.480  (0.039−16.101) | 0.732 (0.028−18.970) | 0.109 |
| **RV16**  **high** | 2.982 (0.042−20.893) | 3.397  (0.045−37.219) | **<0.001** | 3.077 (0.386−18.726) | 3.882 (0.602−37.219) | **0.010** | 0.841 (0.042−20.893) | 0.912 (0.045−22.549) | 0.346 |

***Table S5. Effect of DPM exposure on protein concentrations of chemo- and cytokines (Luminex).*** *Data are presented as median (range) of the protein concentration. Total N=61, children N=49, adults N=12. *p<0.05, tested with Wilcoxon rank-sum test.*

|  | **All** | | | **Children** | | | **adults** | | |
| --- | --- | --- | --- | --- | --- | --- | --- | --- | --- |
| **IFN-γ** | **no diesel** | **diesel** | **p-value** | **no diesel** | **diesel** | **p-value** | **no diesel** | **diesel** | **p-value** |
| **control** | 1.4  (0.0− 8.35) | 1.26  (0.0−11.27) | 0.112 | 1.23  (0.0−5.48) | 1.26  (0.7−9.75) | 0.091 | 4.09  (0.0−8.35) | 2.60  (0.0−11.27) | 0.844 |
| **pI:C** | 1.57 (0.0−12.74) | 1.83  (0.0−15.63) | **0.003** | 1.57  (0.16−11.14) | 1.75  (0.0−14.33) | 0.063 | 2.47  (0.0−12.74) | 3.93  (0.0−15.63) | **0.022** |
| **RV16**  **Low** | 1.26  (0.0−9.6) | 1.71  (0.0−11.21) | **0.015** | 1.25  (0.0−9.6) | 1.71  (0.0−11.21) | **0.039** | 3.76  (0.16−7.01) | 3.69  (0.64−10.16) | 0.184 |
| **RV16**  **high** | 1.59  (0.0−11.14) | 2.10  (0.0−16.7) | **<0.001** | 1.38  (0.0−7.09) | 2.02  (0.0−13.04) | **<0.001** | 2.4  (0.0−11.14) | 3.85  (0.0−16.7) | **0.014** |
| **CXCL10** | **no diesel** | **diesel** | **p-value** | **no diesel** | **diesel** | **p-value** | **no diesel** | **diesel** | **p-value** |
| **control** | 2696.80  (128.31− 34289.39) | 1460.6  (3.21− 17275.01) | **0.002** | 1892.79  (128.31− 25941.95) | 1460.6 (3.21− 17275.01) | **0.003** | 3408.265 (131.55− 34289.39) | 3272.44  (188.476− 15529.04) | 0.239 |
| **pI:C** | 14513.33  (87.89− 36729.33) | 14709.83  (0.016− 59296.9) | 0.244 | 14356.77  ( 87.9− 31243.97) | 14709.83 (143.36− 59296.9) | 0.532 | 16986.7 (87.899− 36729.33) | 15040.84  (143.36− 33795.76) | 0.169 |
| **RV16**  **Low** | 13127.5 (236.47− 25468.24) | 13329.66 (0.011− 28664.18) | 0.994 | 13275.74 (236.47− 25173.12) | 13304.11 (221.49− 18753.37) | 0.739 | 12574.7 (236.47− 25468.24) | 14054.31  (281.76− 28664.18) | 0.477 |
| **RV16**  **high** | 14761.62 (266.69− 30060.2) | 13677.11  (0.012− 30865.35) | 0.272 | 14761.62 (266.69− 27099.14) | 13623.75 (456.86− 30865.35) | 0.471 | 14765.49  (749.95− 30060.2) | 14747.31 (1431.126− 27764.99) | 0.286 |
| **IL-6** | **no diesel** | **diesel** | **p-value** | **no diesel** | **diesel** | **p-value** | **no diesel** | **diesel** | **p-value** |
| **control** | 51.62  (0.0− 2169.07) | 97.51  (0.0− 5258.67) | **<0.001** | 44.6  (0.0− 1703.39) | 57.86  (0.0− 4937.33) | **<0.001** | 174.38 (27.51− 2169.07) | 189.54  (39.20− 5258.67) | 0.136 |
| **pI:C** | 86.71  (0.0− 7433.69) | 246.22 (0.77− 9613.27) | **0.001** | 77.8  (0.0− 7433.69) | 204  (0.77− 5908.47) | **0.005** | 500.2  (41.09− 3951.25) | 672.31 (53.63−9613.27) | 0.139 |
| **RV16**  **Low** | 52.06 (0.11− 4766.55) | 167.65 (0.39− 4869.23) | **<0.001** | 39.95  (0.11− 4766.55) | 154.67  (0.39− 3857.51) | **<0.001** | 99.03  (26.69− 2622.6) | 352.94 (45.228−4869.23) | **0.010** |
| **RV16**  **high** | 78.33  (0.26− 4053.77) | 188.39 (0.89− 7617.01) | **<0.001** | 76.61  (0.26− 4053.77) | 104.55  (0.89− 6829.57) | **<0.001** | 122.31  (33.39− 2362.41) | 332.27  (49.24−7617.01) | 0.075 |
| **CXCL8** | **no diesel** | **diesel** | **p-value** | **no diesel** | **diesel** | **p-value** | **no diesel** | **diesel** | **p-value** |
| **control** | 7246.27 (1124.81− 62300.24) | 10821.48  (0.43− 49015.83) | **0.019** | 7211.02 (1124.81− 62300.24) | 10821.48  (0.43− 44526.81) | **0.027** | 9002.064 (2549.3− 25841.33) | 9192.24  (1961.51− 49015.83) | 0.480 |
| **pI:C** | 10333.85 (1498.08− 69955.64) | 11360.83  (2911.15− 66043.86) | **0.017** | 9137.81  (1498.08− 69955.64) | 10829.15  (2911.15− 66043.86) | **0.004** | 15184.91  (2627.93− 30898.99) | 14036.04 (3393.78− 32677.14) | 0.646 |
| **RV16**  **Low** | 7966.71  (1646.21− 40829.73) | 8848.12 (1732.17− 33055.81) | **0.004** | 8033.65  (1646.21−40829.73) | 9333.65 (1732.17− 33055.81) | **0.009** | 7837.85  (1988.62− 28659.81) | 7465.42  (3502.7− 25392.3) | 0.248 |
| **RV16**  **high** | 8612.46  (1341.79− 119418.9) | 9458.23  (2056.44− 278526.2) | **<0.001** | 8975.83  (1341.79−119418.9) | 9352.66 (2056.44− 278526.2) | **<0.001** | 8177.6 (1646.21− 26990.28) | 11859.81 (3167.21− 23516.16) | 0.656 |

***Table S6. Comparison of the DPM exposure effect on viral titers (TCID50) between children and adults.*** *Data are presented as median (range) of the TCID50 dose normalized to the corresponding non-DPM control.* *Total N=61, children N=49, adults N=12. *p<0.05, tested with Wilcoxon rank-sum test.*

|  | **children** | **adults** | **p-value** |
| --- | --- | --- | --- |
| **RV1b low** | 1.00 (0.01−100) | .10 (0.1−10) | 0.069 |
| **RV1b high** | 1.00 (0.1−10000) | 10.00 (0.1−100) | 0.307 |
| **RV16 Low** | 10.00 (0.01−1.00000) | 10.00 (0.1−1000) | 0.928 |
| **RV16 high** | 10.00 (0.01−10000) | 1.00 (0.1−100) | 0.114 |

***Table S7 (part 1). Comparison of the DPM exposure effect on viral loads and immune factors between children and adults (RT-PCR).*** *Data are presented as median (range) of the mRNA levels normalized to the housekeeping gene PGK1 and to the corresponding non-DPM control.* *Total N=60, children N=48, adults N=12. *p<0.05, tested with Wilcoxon rank-sum test.*

| **RV-load** | **children** | **adults** | **p-value** |
| --- | --- | --- | --- |
| **RV1b low** | 0.925 (0.047−66.38) | 1.148 (0.036−9.651) | 0.682 |
| **RV1b high** | 1.180 (0.076−20.112) | 1.768 (0.401−35.964) | 0.223 |
| **RV16 low** | 1.713 (0.115−67202) | 1.566 (0.207−6.033) | 0.445 |
| **RV16 high** | 1.670 (0.111−56.523) | 2.483 (0.310−9.209) | 0.265 |
| **ICAM-1** | **children** | **adults** | **p-value** |
| **control** | 0.908 (0.169−6.971) | 0.810 (0.424−4.332) | 0.395 |
| **pI:C** | 0.981 (0.240−2.529) | 0.862 (0.430−4.784) | 0.856 |
| **RV1b low** | 0.957 (0.164−1.605) | 0.801 (0.490−1.135) | 0.119 |
| **RV1b high** | 0.885 (0.0245−1.874) | 1.020 (0.351−4.784) | 0.521 |
| **RV16 low** | 0.950 (0.316−3.157) | 0.908 (0.538−1.369) | 0.521 |
| **RV16 high** | 0.935 (0.224−3.529) | 0.882 (0.535−1.023) | 0.377 |
| **LDLR** | **children** | **adults** | **p-value** |
| **control** | 1.130 (0.408−2.786) | 1.075 (0.408−5.009) | 0.367 |
| **pI:C** | 1.158 (0.505−1.91) | 1.430 (0.616−6.668) | 0.112 |
| **RV1b low** | 1.269 (0.544−2.234) | 1.102 (0.772−1.758) | 0.234 |
| **RV1b high** | 1.167 (0.831−1.903) | 1.067 (0.799−6.668) | 0.580 |
| **RV16 low** | 1.225 (0.786−2.550) | 1.173 (0.847−1.528) | 0.275 |
| **RV16 high** | 1.261 (0.418−2.790) | 1.157(0.694−2.294) | 0.628 |
| **TLR3** | **children** | **adults** | **p-value** |
| **control** | 0.798 (0.343−1.850) | 0.541 (0.109−5.084) | 0.318 |
| **pI:C** | 0.906 (0.587−2.570) | 0.932 (0.438−3.080) | 0.666 |
| **RV1b low** | 0.129 (0.382−1.853) | 0.824 (0.319−1.898) | 0.166 |
| **RV1b high** | 0.928 (0.417−1.491) | 0.716 (0.232−1.223) | 0.177 |
| **RV16 low** | 0.968 (0.082−1.817) | 0.829 (0.033−1.653) | 0.199 |
| **RV16 high** | 0.980 (0.424−7.201) | 0.731 (0.381−1.547) | 0.073 |
| **MDA5** | **children** | **adults** | **p-value** |
| **control** | 0.789 (0.262−1.291) | 0.566 (0.188−16.481) | 0.416 |
| **pI:C** | 1.037 (0.514−3.792) | 1.000 (0.276−9.933) | 0.554 |
| **RV1b low** | 0.905 (0.375−1.309) | 0.650 (0.289−1.269) | 0.152 |
| **RV1b high** | 0.894 (0.333−2.631) | 0.733 (0.398−2.078) | 0.120 |
| **RV16 low** | 1.099 (0.318−5.147) | 0.803 (0.209−5.281) | 0.094 |
| **RV16 high** | 1.071 (0.390−2.638) | 0.748 (0.293−5.688) | 0.155 |
| **RIG-1** | **children** | **adults** | **p-value** |
| **control** | 0.770 (0.268−1.460) | 0.619 (0.157− 23.856) | 0.160 |
| **pI:C** | 0.945 (0.353−4.545) | 0.931 (0.240−17.100) | 0.666 |
| **RV1b low** | 0.873 (0.219−1.4112) | 0.683 (0.268−1.300) | 0.145 |
| **RV1b high** | 0.799 (0.244−3.369) | 0.685 (0.451−2.265) | 0.458 |
| **RV16 low** | 1.123 (0.314−4.085) | 0.706 (0.293−1.583) | **0.028** |
| **RV16 high** | 1.146 (0.377−4.172) | 0.719 (0.373−4.7120) | **0.035** |
| **βDEF-2** | **children** | **adults** | **p-value** |
| **control** | 1.380 (0.044−23.409) | 0.834 (0.1495−11.311) | 0.216 |
| **pI:C** | 1.397 (0.124−17.436) | 1.283 (0.215−20.706) | 0.813 |
| **RV1b low** | 1.772 (0.005−14.603) | 1.074 (0.300−5.764) | 0.174 |
| **RV1b high** | 1.187 (0.219−5.357) | 0.998 (0.265−5.002) | 0.326 |
| **RV16 low** | 1.857 (0.223−18.226) | 1.199 (0.170−2.357) | **0.016** |
| **RV16 high** | 1.29 (0.1706−6.901) | 0.999 (0.082−2.625) | 0.181 |
| **IFN-β** | **children** | **adults** | **p-value** |
| **control** | 0.969 0.001−7.131) | 0.466 (0.220−2.883) | 0.073 |
| **pI:C** | 0.869 (0.157−112.868) | 0.808 (0.128−21.156) | 0.849 |
| **RV1b low** | 0.972 (0.010−32.469) | 0.680 (0.124−1.236) | 0.506 |
| **RV1b high** | 0.941 (0.2160−25.767) | 0.566 (0.396−2.079) | 0.225 |
| **RV16 low** | 1.413 (0.106−60.317) | 0.979 (0.334−6.577) | 0.260 |
| **RV16 high** | 1.105 (0.061−14.462) | 1.436 (0.340−50.823) | 0.237 |

***Table S7 (part 2). Comparison of the DPM exposure effect on viral loads and immune factors between children and adults (RT-PCR).*** *Data are presented as median (range) of the mRNA levels normalized to the housekeeping gene PGK1 and to the corresponding non-DPM control.* *Total N=60, children N=48, adults N=12. *p<0.05, tested with rank-sum Wilcoxon test.*

| **IFN-λ** | **children** | **adults** | **p-value** |
| --- | --- | --- | --- |
| **control** | 0.954(0.536−1.517) | 0.820 (0.311−4.59) | 0.243 |
| **pI:C** | 1.028 (0.548−2.712) | 1.014 (0.658−4.555) | 0.909 |
| **RV1b low** | 0.984 (0.324−1.434) | 0.850 (0.449−1.66) | **0.036** |
| **RV1b high** | 0.982 (0.589−1.681) | 1.001 (0.619−4.555) | 0.989 |
| **RV16 low** | 0.926 (0.615−2.331) | 0.966 (0.378−1.441) | 0.952 |
| **RV16 high** | 0.973 (0.453−2.137) | 0.910 (0.336−1.817) | 0.420 |
| **CXCL10** | **children** | **adults** | **p-value** |
| **control** | 0.551 (0.037−3.510) | 0.254 (0.049−64.251) | 0.116 |
| **pI:C** | 0.965 (0.040−26.946) | 1.095 (0.1300−164.772) | 0.802 |
| **RV1b low** | 0.825 (0.011−19.346) | 0.592 (0.113−1.107) | 0.198 |
| **RV1b high** | 0.612 (0.089−16.223) | 0.309 (0.145−2.083) | 0.226 |
| **RV16 low** | 1.222 (0.091−72.178) | 0.609 (0.139−2.437) | 0.284 |
| **RV16 high** | 0.887 (0.042−10.026) | 0.441 (0.003−6.090) | **0.035** |
| **IL-1β** | **children** | **adults** | **p-value** |
| **control** | 1.543 (0.051−8.963) | 1.476 (0.604−3.071) | 0.969 |
| **pI:C** | 2.084 (0.276−18.584) | 2.053 (1.122−2.395) | 0.678 |
| **RV1b low** | 2.021 (0.153−4.359) | 1.763 (0.589−3.495) | 0.213 |
| **RV1b high** | 1.951 (0.511−9.462) | 1.763 (0.778−2.746) | 0.658 |
| **RV16 low** | 1.953 (0.491−27.189) | 2.123 (0.540−4.481) | 0.737 |
| **RV16 high** | 1.768 (0.259−11.382) | 2.151 (0.704−4.314) | 0.599 |
| **IL-6** | **children** | **adults** | **p-value** |
| **control** | 1.365 (0.103−4.446) | 1.699 (0.293−15.721) | 0.127 |
| **pI:C** | 1.456 (0.141−15.654) | 1.880 (0.524−12.478) | 0.097 |
| **RV1b low** | 2.032 (0.115−7.631) | 1.615 (0.346−2.896) | 0.154 |
| **RV1b high** | 1.474 (0.186−3.955) | 1.529 (0.814−12.478) | 0.408 |
| **RV16 low** | 1.419 (0.278−15.038) | 1.527 (0.573−4.991) | 0.572 |
| **RV16 high** | 1.050 (0.001−7.291) | 1.278 (0.421−22.225) | 0.249 |
| **CXCL8** | **children** | **adults** | **p-value** |
| **control** | 1.401 (0.154−2.409) | 1.308 (0.446−2.409) | 0.486 |
| **pI:C** | 1.505 (0.636−5.254) | 2.170 (0.494−5.254) | 0.437 |
| **RV1b low** | 1.482 (0.124−2.158) | 1.185 (0.446−2.158) | 0.221 |
| **RV1b high** | 1.603 (0.440−2.546) | 1.279 (0.446−2.546) | 0.503 |
| **RV16 low** | 1.527 (0.582−3.116) | 1.228 (0.482−3.116) | 0.307 |
| **RV16 high** | 1.341 (9.334−3.454) | 1.277 (0.501−3.454) | 0.489 |

***Table S8. Comparison of the DPM exposure effect on protein levels between children and adults (Luminex).*** *Data are presented as median (range) of the protein concentration normalized to the corresponding non-DPM control.* *Total N=61, children N=49, adults N=12. *p<0.05, tested with rank-sum Wilcoxon test.*

| **IFN-γ** | **children** | **adults** | **p-value** |
| --- | --- | --- | --- |
| **control** | 1.1329 (0.000−9.571) | 1.1055 (0.032−2.300) | 0.577 |
| **pI:C** | 1.045 (0.000−9.571) | 1.498 (0.910−4.487) | 0.078 |
| **RV16 low** | 1.118 (0.000−9.571) | 1.220 (0.000−2.513) | 0.911 |
| **RV16 high** | 1.267 (0.000−14.142) | 1.382 (0.465−2.864) | 0.309 |
| **IL-6** | **children** | **adults** | **p-value** |
| **control** | 1.860 (0.000−12.161) | 1.712 (0.154−4.153) | 0.540 |
| **pI:C** | 1.888 (0.420−11.447) | 1.477 (0.471−3.020) | 0.492 |
| **RV16 low** | 1.863 (0.194−39.426) | 1.894 (0.707−5.116) | 0.508 |
| **RV16 high** | 1.874 (0.032−9.165) | 2.301 (0.688−8.738) | 0.741 |
| **IL-8** | **children** | **adults** | **p-value** |
| **control** | 1.332 (0.000−5.97) | 0.939 (0.182−1.253) | 0.432 |
| **pI:C** | 1.498 (0.170−9.967) | 1.098 (0.259−2.819) | 0.244 |
| **RV16 low** | 1.235 (0.182−5.561) | 1.164 (0.634−1.761) | 0.613 |
| **RV16 high** | 1.476 (0.1485−9.812) | 1.199 (0.472−2.580) | 0.613 |
| **IP-10** | **children** | **adults** | **p-value** |
| **control** | 0.906 (0.001−20.778) | 0.758 (0.069−3.272) | 0.732 |
| **pI:C** | 1.007 (0.116−6.668) | 0.9545 (0.610−2.485) | 0.692 |
| **RV16 low** | 1.021 (0.069−8.576) | 1.103 (0.089−36.821) | 0.712 |
| **RV16 high** | 0.973 (0.047−3.950) | 0.923 (0.140−24.930) | 0.425 |

# References

1. Schogler A, Kopf BS, Edwards MR, Johnston SL, Casaulta C, Kieninger E, Jung A, Moeller A, Geiser T, Regamey N, et al. Novel antiviral properties of azithromycin in cystic fibrosis airway epithelial cells. *The European respiratory journal* 2015;45(2):428-439.

2. Gielen V, Johnston SL, Edwards MR. Azithromycin induces anti-viral responses in bronchial epithelial cells. *The European respiratory journal* 2010;36(3):646-654.
